# Impression-regularized finetuning enables small language models to extract cervical spine fractures from radiology reports

**DOI:** 10.1371/journal.pdig.0001786

**Published:** 2026-10-21

**Authors:** Zixuan Hu, Hui Ming Lin, Yingming Amy Chen, Christopher S. Lozano, Markand Patel, Ali Emami, Ervin Sejdić, Errol Colak

**Affiliations:** 1 Department of Electrical and Computer Engineering, University of Toronto, Toronto, Ontario, Canada; 2 Department of Medical Imaging, St Michael’s Hospital, Unity Health Toronto, Toronto, Ontario, Canada; 3 Department of Medical Imaging, Faculty of Medicine, University of Toronto, Toronto, Ontario, Canada; 4 Division of Neurosurgery, Department of Surgery, University of Toronto, Toronto, Ontario, Canada; 5 Department of Computer Science, Emory University, Atlanta, Georgia, United States of America; 6 Research and Innovation, North York General Hospital, Toronto, Ontario, Canada; 7 Li Ka Shing Knowledge Institute, St Michael’s Hospital, Unity Health Toronto, Toronto, Ontario, Canada; The Second Affiliated Hospital of Chongqing Medical University, CHINA

## Abstract

We assessed whether small, open-weight large language models (LLMs) can accurately extract vertebral-level acute cervical spine fracture labels from CT radiology reports while supporting on-premises deployment, and whether impression-regularized finetuning improves accuracy, data efficiency, and temporal stability. In this single-center retrospective study, 8,567 adult cervical spine CT reports (2017–2022) were labeled for acute occipital condyle and C1–C7 fractures with neuroradiologist oversight. Reports from 2017 (n = 1,064) were used for finetuning and reports from 2018–2022 (n = 7,503) for temporal testing. We finetuned Gemma-3 (1B/4B/12B), Llama-3.2-3B, and Phi-3.5-Mini using standard low-rank adaptation (LoRA) and with an impression-regularized LoRA approach. During training, the model samples multiple synthetic targeted impressions focused on acute cervical spine fractures, selects the candidate most consistent with the reference standard labels, and applies an auxiliary impression loss as regularization. At inference, all models perform single-pass deterministic JSON extraction. Baselines included zero-shot prompting, two-pass impression-guided prompting, and standard LoRA. Impression-regularized LoRA improved Gemma-3-4B macro-F1 from 0.885 to 0.909 (macro-precision 0.929, macro-recall 0.893) while reducing variance across training runs. Improvements were largest for smaller models and in low-data settings; Gemma-3-1B macro-F1 increased from 0.646 to 0.782. For this vertebral-level acute cervical spine fracture extraction task, impression-regularized Gemma-3-4B approached the zero-shot performance of the Gemma-3-27B (macro-F1 0.909 vs. 0.913). Across the 2018–2022 temporal evaluation, mean F1 increased from 0.871 to 0.893 with similar or lower variability across time windows. Systematic error analysis showed that remaining errors were mainly associated with ambiguous report language and label-boundary cases. Overall, impression-regularized LoRA enables small open-weight LLMs to accurately extract acute cervical spine fracture labels while maintaining performance across a multi-year test period and preserving auditable single-pass inference suitable for privacy-preserving on-premises deployment.

## Author summary

Radiology reports contain important details about injuries, but these details are often written in free text rather than in a format that can be easily searched, counted, or used for quality improvement. In this study, we examined whether smaller language models can extract structured information about acute cervical spine fractures from computed tomography reports, including which specific anatomic level is involved. Our approach trains the model not only to produce the final structured labels, but also to learn from a short, report-like summary of the relevant findings during training. This added supervision is not intended to be a clinical explanation or a replacement for radiologist review. Instead, it provides an additional learning signal that may help the model focus on fracture location, acuity, and negation. We found that this approach improved performance in our single-center dataset, particularly for smaller models and limited training data. Performance was also maintained across reports from later years in the same institution. These findings suggest that locally deployable language models may help support retrospective cohort identification, monitoring, or non-blocking workflow tools. However, external validation, prospective monitoring, and careful attention to errors and false alerts are needed before broader clinical use.

## 1. Introduction

Automated extraction of structured labels from radiology reports is a key enabler for scalable medical imaging AI [1]. Large-scale model development, retrospective cohort assembly, and post-deployment monitoring all depend on reliable, machine-readable outcomes, but radiology reports are often free-text narratives and linguistically complex, often combining findings with negation, uncertainty, and historical context, and may describe multiple anatomical regions within a single report [2,3]. These properties make both manual abstraction and automated parsing error-prone, particularly for safety-critical tasks such as fracture detection [1,4].

We study cervical spine fracture labeling as a representative and clinically consequential test case. Cervical spine computed tomography (CT) is widely used in trauma, where missed fractures carry substantial neurologic risk and false positives may lead to over-triage, transfer to a trauma or spine center, and unnecessary neurosurgical or orthopaedic spine consultation [5–7]. Our label schema includes occipital condyle and C1–C7 fractures. Occipital condyle fractures involve the craniocervical junction and may be associated with craniovertebral instability [8], while C1–C7 labels localize injury across the cervical spine. By retaining the injured vertebral level, this schema preserves location information that informs radiologic interpretation and trauma triage while enabling level-specific cohort construction and model evaluation. From a language modeling perspective, reports must distinguish acute traumatic injuries from chronic or degenerative changes, interpret exclusionary phrasing, assign the correct anatomic level, and ignore unrelated findings from adjacent regions such as the skull base, thoracic spine, or lung apices [9–11].

Traditional NLP pipelines for fracture labeling have relied on task-specific models trained on thousands of annotated reports to reach moderate-to-high performance [3,4]. More recent work using LLMs has demonstrated improved accuracy with less supervision, but often depends on very large models, complex prompting strategies, and limited temporal evaluation, limiting practical deployment [12–18]. For radiology departments, the critical barriers are not just performance, but requirements for on-premises deployment, data privacy, computational efficiency, and auditability [19,20]. Small, open-weight LLMs address these needs by enabling local execution and predictable costs. However, direct report-to-label extraction remains difficult for smaller models under limited supervision, as they must simultaneously master nuanced clinical semantics and rigid structured output [21,22].

A natural strategy is to decompose this joint task with an intermediate representation between the report and the final labels [23,24], separating clinical interpretation from the generation of rigid structured output. Radiology reports already contain a domain-native intermediate form, “the impression”, which professional reporting guidelines recommend as a concise synthesis of diagnostic conclusions [25,26]; although its wording and structure vary across radiologists and institutions, this shared function makes it a natural intermediate target. Prior work has shown that LLMs can generate impressions with high completeness and correctness. However, impression-only pipelines introduce a hard bottleneck: fracture-relevant details may be omitted or obscured among unrelated findings, particularly when multiple anatomical regions are described, and any upstream error directly propagates to the final labels. In addition, generating impressions at inference time increases computational cost and introduces additional variability [27,28].

In this work, we leverage impressions differently. We define targeted impressions as concise summaries restricted to acute cervical spine fracture–relevant findings and use them only during training. Motivated by evidence that sampling multiple candidate generations and selecting those favored by a correctness signal can improve LLM performance [29–31], we introduce impression-regularized finetuning, which generates multiple candidate targeted impressions per report, scores them by consistency with expert labels, and distills the most label-consistent impression as auxiliary supervision. This design provides an auxiliary, fracture-focused text target during training without requiring expert-annotated chains-of-thought and preserves simple single-pass JSON extraction at deployment.

We instantiate this idea for vertebral-level acute cervical spine fracture labeling and make three contributions:

1. Introduce impression-regularized LoRA, a finetuning framework that uses targeted, task-focused radiology impressions as latent supervision while preserving simple, auditable single-pass JSON inference.
2. Systematically compare impression-regularized LoRA to zero-shot prompting, two-pass impression-guided prompting, and standard JSON-only LoRA, across multiple small open-weight LLMs and training-set sizes, measuring vertebral-level performance and multi-year temporal stability.
3. Demonstrate that, for vertebral-level acute cervical spine fracture labeling, a 4B-parameter model finetuned with impression-regularized LoRA achieves high accuracy and maintains performance across a multi-year test period, approaching the performance of a 27B-parameter zero-shot baseline.

## 2. Results

### 2.1. Baseline performance across model families

Zero-shot single-pass JSON extraction produced macro-F1 scores of 0.669 for Gemma-3-4B, 0.791 for Llama-3.2-3B, and 0.859 for Phi-3.5-Mini (Table 1). Overall, Phi-3.5-Mini provided the strongest zero-shot baseline, followed by Llama-3.2-3B, while Gemma-3-4B trailed in direct extraction performance.

**Table 1:** Extraction performance for Gemma-3-4B, Llama-3.2-3B, and Phi-3.5-Mini under zero-shot prompting and LoRA finetuning, with and without impression-regularized supervision, reported as macro-F1, macro-precision, and macro-recall on the held-out 2018-2022 test set. Bolded values represent the highest mean value observed for each metric within each model.

Model Variant	Method	Macro-F1	Macro Precision	Macro Recall	Report Level Sensitivity	Report Level Specificity
Gemma-3	Zero-shot (Direct)	0.669	0.543	0.932	**0.968**	0.934
	Zero-shot (Impression-guided)	0.730	0.616	**0.938**	**0.968**	0.959
	LoRA (Standard)	0.885 ± 0.022	0.883 ± 0.062	0.899 ± 0.028	0.937 ± 0.025	0.987 ± 0.010
	LoRA (Impression-regularized)	**0.909 ± 0.004**	**0.929 ± 0.014**	0.893 ± 0.010	0.943 ± 0.014	**0.995 ± 0.002**
Llama-3.2	Zero-shot (Direct)	0.791	0.747	0.856	0.889	0.983
	Zero-shot (Impression-guided)	0.797	0.776	0.836	0.905	0.982
	LoRA (Standard)	0.890 ± 0.004	0.918 ± 0.024	0.867 ± 0.021	0.918 ± 0.012	0.993 ± 0.003
	LoRA (Impression-regularized)	**0.902 ± 0.004**	**0.937 ± 0.022**	**0.874 ± 0.020**	**0.924 ± 0.018**	**0.995 ± 0.003**
Phi-3.5	Zero-shot (Direct)	0.859	0.885	0.845	**0.898**	0.992
	Zero-shot (Impression-guided)	0.763	**0.938**	0.668	0.760	0.993
	LoRA (Standard)	0.877 ± 0.008	**0.938 ± 0.011**	0.827 ± 0.018	0.880 ± 0.011	**0.996 ± 0.000**
	LoRA (Impression-regularized)	**0.880 ± 0.003**	0.919 ± 0.004	**0.848 ± 0.006**	0.897 ± 0.004	0.995 ± 0.000

Two-pass impression-guided prompting improved macro-F1 for Gemma-3-4B (0.669 to 0.730) and slightly improved Llama-3.2-3B (0.791 to 0.797) but substantially reduced Phi-3.5-Mini (0.859 to 0.763). For Gemma and Llama, the gains were primarily precision-driven with comparable recall, whereas Phi’s two-pass setting increased precision but incurred a pronounced recall drop, leading to worse overall macro-F1 (Table 1).

At the report-level, zero-shot configurations already achieved high specificity (0.934 to 0.992) but variable sensitivity (0.889 to 0.968). Two-pass prompting modestly increased specificity for Gemma-3-4B and maintained similar specificity for Llama-3.2-3B (0.959 and 0.982, respectively) while keeping sensitivity similar, whereas Phi-3.5-Mini showed reduced report-level sensitivity under two-pass prompting (Table 1).

### 2.2. Fine-tuned performance and data efficiency

Across all model families, LoRA finetuning substantially improved vertebral-level extraction performance relative to zero-shot baselines, and impression-regularized LoRA provided additional gains (Table 1, Fig 1). When trained using the full 2017 training cohort, impression-regularized LoRA increased macro-F1 for Gemma-3-4B from 0.885 ± 0.022 to 0.909 ± 0.004 (p = 0.002; here and throughout, statistical comparisons between impression-regularized LoRA and standard LoRA use a hierarchical paired bootstrap over random seeds and test cases, with details provided in Section 4.9) and for Llama-3.2-3B from 0.890 ± 0.004 to 0.902 ± 0.004 (p = 0.002), while Phi-3.5-Mini improved only marginally from 0.877 ± 0.008 to 0.880 ± 0.003 (p = 0.432). These gains were largely explained by improved macro-precision with similar macro-recall, and report-level specificity remained high (≥ 0.993) with small sensitivity increases (Table 1).

**Table 2:** Scaling behavior within the Gemma-3 family (1B, 4B, and 12B parameters) under zero-shot prompting and LoRA finetuning, with and without impression-regularized supervision, while Gemma-3-27B was included as a zero-shot baseline. Metrics are reported as macro-F1, macro-precision and macro-recall on the held-out 2018-2022 test set. Bolded values represent the highest mean value observed for each metric within each model.

Model Size	Method	Macro-F1	Macro Precision	Macro Recall	Report Level Sensitivity	Report Level Specificity
1B	Zero-shot (Direct)	0.215	0.480	0.158	0.183	0.990
	LoRA (Standard)	0.646 ± 0.023	0.833 ± 0.017	0.553 ± 0.024	0.685 ± 0.030	0.990 ± 0.001
	LoRA (Impression-regularized)	**0.782 ± 0.023**	**0.879 ± 0.028**	**0.713 ± 0.032**	**0.794 ± 0.025**	**0.994 ± 0.001**
4B	Zero-shot (Direct)	0.669	0.543	**0.932**	**0.968**	0.934
	LoRA (Standard)	0.885 ± 0.022	0.883 ± 0.062	0.899 ± 0.028	0.937 ± 0.025	0.987 ± 0.010
	LoRA (Impression-regularized)	**0.909 ± 0.004**	**0.929 ± 0.014**	0.893 ± 0.010	0.943 ± 0.014	**0.995 ± 0.002**
12B	Zero-shot (Direct)	0.901	0.903	**0.907**	**0.925**	0.994
	LoRA (Standard)	0.921 ± 0.006	0.944 ± 0.016	0.900 ± 0.017	0.921 ± 0.025	0.996 ± 0.002
	LoRA (Impression-regularized)	**0.925 ± 0.003**	**0.953 ± 0.008**	0.899 ± 0.007	0.914 ± 0.008	**0.998 ± 0.000**
27B	Zero-shot (Direct)	0.913	0.908	0.922	0.934	0.995

**Fig 1:**
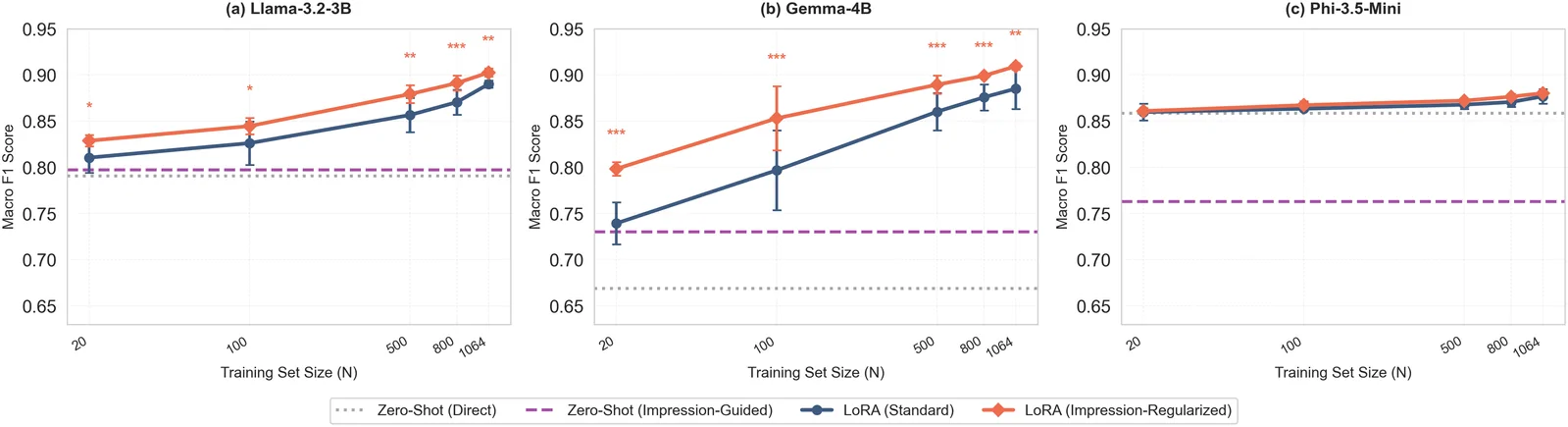
Cross-model learning curves for vertebral-level acute fracture extraction. Macro-F1 (mean ± SD over five independent runs) is shown as a function of finetuning set size (N = 20, 100, 500, 800, 1064) for **(a)** Llama-3.2-3B, **(b)** Gemma-3-4B, and **(c)** Phi-3.5-Mini. Dashed horizontal lines denote zero-shot baselines using single-pass JSON extraction and two-pass impression-guided prompting. Solid curves show LoRA finetuning with direct JSON supervision and impression-regularized supervision. Points indicate run means, and error bars represent ±1 standard deviation. Statistical significance between impression-regularized and standard LoRA results at each training size is indicated by * (p < 0.05), ** (p < 0.01), and *** (p < 0.001).

In the data-efficiency setting (Fig 1; Table M in S1 Appendix), impression-regularized LoRA showed its advantage in the smallest-data regimes and generally converged toward the standard-LoRA baseline as more labeled reports were added. Gemma-3-4B exhibited the largest low-data gains, improving from 0.739 to 0.798 at N = 20 (+0.059, p < 0.001) and from 0.797 to 0.853 at N = 100 (+0.056, p < 0.001), with a smaller but still significant advantage at the full 2017 set (0.885 to 0.909; p = 0.002). Llama-3.2-3B started from a higher baseline and showed moderate but consistent improvements at low N (0.810 to 0.829 at N = 20, p = 0.046; 0.826 to 0.845 at N = 100, p = 0.018) while maintaining a similar margin at larger training sizes. Phi-3.5-Mini showed a different pattern: it began with the strongest zero-shot and low-data baselines, and impression-regularized LoRA produced only small, non-significant changes across training sizes. In the full-data setting, standard LoRA and impression-regularized LoRA reached similar macro-F1 but different precision–recall profiles, with standard LoRA achieving higher macro-precision (0.938 vs. 0.919) and impression regularization slightly improving macro-recall (0.827 to 0.848).

### 2.3. Scaling behavior within Gemma

Within the Gemma-3 family, impression-regularized LoRA improved macro-F1 at all evaluated scales, but with strongly scale-dependent gains (Table 2, Fig 2). The largest improvement occurred for Gemma-3-1B, where macro-F1 increased from 0.646 ± 0.023 to 0.782 ± 0.023 (p < 0.001). For Gemma-3-4B, macro-F1 increased from 0.885 ± 0.022 to 0.909 ± 0.004 (p = 0.002). For Gemma-3-12B, which was already near ceiling after standard LoRA, the incremental improvement was small (0.921 ± 0.006 to 0.925 ± 0.003; p = 0.254).

**Fig 2:**
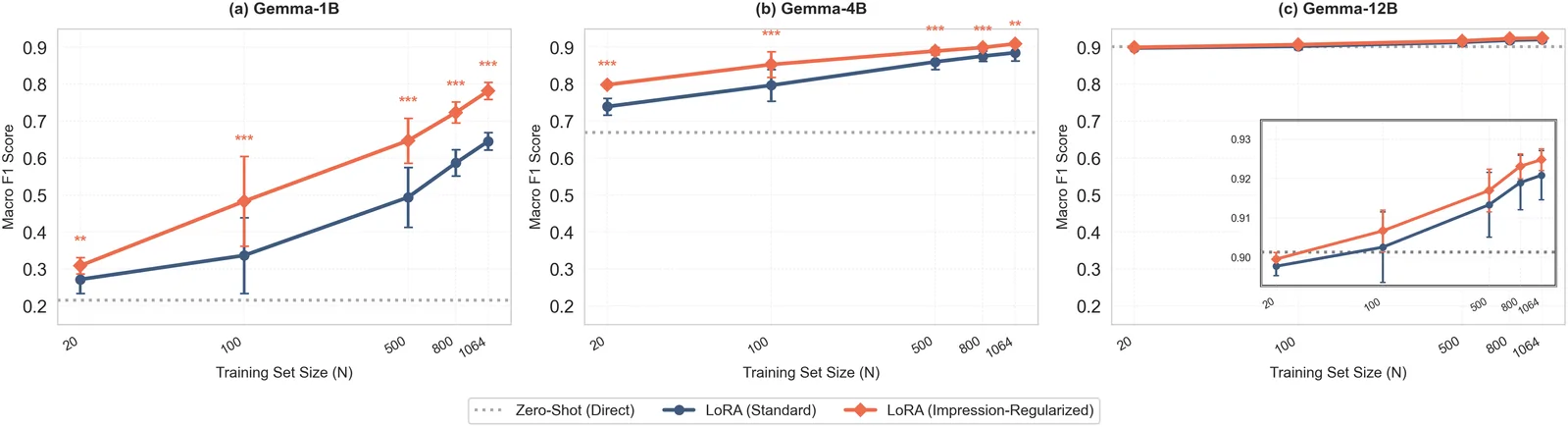
Scaling of learning curves within the Gemma model family. Macro-F1 (mean ± SD over five independent runs) is shown as a function of finetuning set size (N = 20, 100, 500, 800, 1064) for **(a)** Gemma-3-1B, **(b)** Gemma-3-4B, and **(c)** Gemma-3-12B, comparing LoRA finetuning with direct JSON supervision and impression-regularized supervision. Dashed horizontal lines indicate zero-shot direct performance within each panel. Points denote run means, and error bars represent ±1 standard deviation. Statistical significance between impression-regularized and direct LoRA results at each training size is indicated by * (p < 0.05), ** (p < 0.01), and *** (p < 0.001).

At the report-level, impression-regularized LoRA generally increased sensitivity while preserving very high specificity (≥ 0.994 across Gemma scales in Table 2). Notably, for this extraction task, Gemma-3-4B with impression-regularized LoRA (macro-F1 0.909; report-level sensitivity/specificity 0.943/0.995) closely approached the performance of Gemma-3-27B in the zero-shot setting (macro-F1 0.913; 0.934/0.995), suggesting that targeted auxiliary supervision can partially offset limited model capacity without sacrificing clinically relevant specificity.

### 2.4. Temporal performance over 2018–2022

Supplementary temporal audits showed that the 2018–2022 test period was not a fully stationary extension of the 2017 training year, with measurable differences in cohort age, fracture prevalence, report length, vocabulary overlap, software-version metadata, and series-description metadata (Table A and Table B in S1 Appendix). On the multi-year 2018–2022 test set, impression-regularized LoRA achieved a higher mean temporal F1 than standard LoRA for every model, with similar or lower window-to-window dispersion (Table 3; Fig A in S1 Appendix). The differences in mean F1 were largest for the smaller Gemma models. Specifically, across 90-day sliding windows, Gemma-3-1B improved its temporal mean from 0.615 ± 0.100 to 0.750 ± 0.084, with coefficient of variation (CV) decreasing from 16.32% to 11.15%, and Gemma-3-4B increased from 0.871 ± 0.062 to 0.893 ± 0.055, with CV decreasing from 7.13% to 6.19% (Table 3).

**Table 3:** Temporal performance of all model families on the 2018–2022 test set. For each model and supervision regime, the table reports temporal mean F1 (± standard deviation across sliding windows) and temporal coefficient of variation (CV, %). Impression-regularized LoRA generally increases temporal F1 and, especially for Gemma-3-1B and Gemma-3-4B, reduces temporal variability, whereas zero-shot impression guidance has mixed effects across models. Bolded values represent the highest mean F1, and lowest CV value observed for each model across methods.

Model	Method	Temporal F1	Temporal CV (%)
Gemma-3-1B	Zero-shot (Direct)	0.199 ± 0.074	37.30
	LoRA (Standard)	0.615 ± 0.100	16.32
	LoRA (Impression-regularized)	**0.750 ± 0.084**	**11.15**
Llama-3.2-3B	Zero-shot (Direct)	0.780 ± 0.083	10.62
	Zero-shot (Impression-guided)	0.777 ± 0.082	10.54
	LoRA (Standard)	0.872 ± 0.063	7.19
	LoRA (Impression-regularized)	**0.886 ± 0.062**	**7.00**
Gemma-3-4B	Zero-shot (Direct)	0.671 ± 0.067	10.00
	Zero-shot (Impression-guided)	0.722 ± 0.070	9.62
	LoRA (Standard)	0.871 ± 0.062	7.13
	LoRA (Impression-regularized)	**0.893 ± 0.055**	**6.19**
Phi-3.5-Mini	Zero-shot (Direct)	0.832 ± 0.069	**8.24**
	Zero-shot (Impression-guided)	0.739 ± 0.083	11.27
	LoRA (Standard)	0.849 ± 0.074	8.76
	LoRA (Impression-regularized)	**0.854 ± 0.074**	8.71
Gemma-3-12B	Zero-shot (Direct)	0.887 ± 0.059	6.60
	LoRA (Standard)	0.904 ± 0.056	**6.18**
	LoRA (Impression-regularized)	**0.909 ± 0.057**	6.26
Gemma-3-27B	Zero-shot (Direct)	0.896 ± 0.053	5.95

For Llama-3.2-3B and Phi-3.5-Mini, impression regularization yielded smaller mean improvements and largely similar temporal variability (Llama: 0.872 ± 0.063 to 0.886 ± 0.062; Phi: 0.849 ± 0.074 to 0.854 ± 0.074), consistent with their higher baselines and smaller low-data headroom. Gemma-3-12B and Gemma-3-27B achieved the highest overall temporal F1, but impression-regularized LoRA narrowed the performance gap for smaller models while preserving, and in some cases improving, temporal stability.

### 2.5. Per-level performance

Per-level precision and recall for Gemma-3-4B on the 2018–2022 test set are summarized in Table 4. With impression-regularized LoRA, precision was ≥ 0.862 and recall was ≥ 0.833 across OCC and C1–C7. The relatively lower values were observed at the mid-cervical levels, with the lowest precision at C3 (0.862 ± 0.009), lower recall at C4 (0.871 ± 0.010), and the lowest recall at C5 (0.833 ± 0.014). Compared with standard LoRA, impression-regularized LoRA generally improved per-level precision while maintaining similar recall, consistent with the precision-driven gains observed at the macro level.

**Table 4:** Per-level precision and recall for Gemma-3-4B on the 2018–2022 temporal test set. For each vertebral level (OCC, C1–C7), precision (P) and recall (R) are reported under zero-shot prompting, standard LoRA, and impression-regularized LoRA. LoRA results are reported as mean ± standard deviation across five seeds, whereas zero-shot results are from a single deterministic run. The number of reference standard-positive reports per level is shown as n positive. Abbreviations: OCC, occipital condyle; P, precision; R, recall.

Level	n positive	Zero-Shot (Direct)		Standard LoRA		Impression-regularized LoRA	
		P	R	P	R	P	R
OCC	118	0.297	0.966	0.687 ± 0.233	0.903 ± 0.065	0.905 ± 0.092	0.905 ± 0.035
C1	95	0.556	0.947	0.956 ± 0.032	0.880 ± 0.030	0.954 ± 0.028	0.880 ± 0.034
C2	186	0.730	0.844	0.938 ± 0.033	0.935 ± 0.024	0.950 ± 0.016	0.945 ± 0.015
C3	71	0.402	0.958	0.874 ± 0.049	0.899 ± 0.012	0.862 ± 0.009	0.899 ± 0.012
C4	84	0.393	0.940	0.897 ± 0.058	0.890 ± 0.026	0.922 ± 0.014	0.871 ± 0.010
C5	134	0.547	0.918	0.886 ± 0.074	0.845 ± 0.059	0.939 ± 0.032	0.833 ± 0.014
C6	162	0.610	0.957	0.904 ± 0.055	0.923 ± 0.027	0.945 ± 0.026	0.914 ± 0.014
C7	217	0.810	0.922	0.926 ± 0.040	0.920 ± 0.029	0.955 ± 0.012	0.895 ± 0.027

### 2.6. Qualitative error patterns

A review of report-level false positives (N = 23) and false negatives (N = 30) revealed recurring error patterns as shown in Table 5. Among the 30 false-negative cases, the most common pattern was missed detection of explicitly described fractures (13/30, 43.3%). Equivocal or ambiguous wording accounted for 9/30 (30.0%) false negatives, complex reports with multiple fracture levels for 5/30 (16.7%), and more implicit wedge-type descriptions without the word “fracture” for 3/30 (10.0%). Of the 23 false-positive cases, 12/23 (52.2%) were due to pathologic or degenerative lesions being misinterpreted as acute fractures, and 9/23 (39.1%) reflected post-surgical or hardware-related changes labeled as fractures. Only two cases were attributable to rarer issues: one anatomical confusion between the occipital bone and occipital condyle (1/23, 4.3%) and one typographical/extraction error (1/23, 4.3%).

**Table 5:** Summary of error mechanisms for false-positive and false-negative reports. The table reports the number of cases and percentage of all reviewed false positives and false negatives assigned to each reason category.

*False Positives*			
Reason category	Count	%	Explanation
Pathologic or degenerative lesion misinterpreted	12	52.2%	Many reports contained metastatic lesions, degenerative osteophytes, or other nonacute pathology. Degenerative changes and nutrient foramina can mimic fractures on CT, leading the AI model to misclassify these findings as fractures.
Post-surgical or hardware related changes	9	39.1%	Prior instrumentation such as spinal fusion or laminoplasty produces defects that may resemble fractures. AI algorithms can misclassify post-surgical changes as fractures.
Anatomical confusion (occipital condyle)	1	4.3%	In at least one case the model confused the occipital bone with the occipital condyle. The occipital condyle is part of the occiput, so imprecise terminology led to mislabelling.
Typographical or extraction error	1	4.3%	A T2 fracture was mistakenly documented as C2 in the report, causing the model to extract the wrong level.
***False Negatives***			
**Reason category**	**Count**	**%**	**Explanation**
Explicit fracture missed	13	43.3%	The report contained a clear, explicit description of an acute fracture, but the model failed to identify the corresponding fracture label, indicating limited sensitivity.
Equivocal or ambiguous wording	9	30.0%	The radiologist used uncertain language such as “equivocal,” “possible,” “may represent,” or other hedging phrases. Ambiguous wording can confuse the AI model; for example, use of terms like “loss of height” or “wedging” is not always recognised as a fracture.
Multiple fractures (complex)	5	16.7%	Reports describing fractures at several levels can overwhelm the model. Complex descriptions of multiple injuries may make it harder for the algorithm to tag every fractured vertebra.
Implicit wording or wedge description	3	10.0%	The radiologist described a deformity or wedge compression but did not explicitly use the word “fracture.” Such terminology (e.g., “wedging”) is ambiguous and may be misinterpreted.

## 3. Discussion

In this single-center cohort, small open-weight LLMs, when trained with impression-regularized LoRA, can accurately extract vertebral-level acute cervical spine fracture labels from free-text CT reports. Using Gemma-3-4B as a representative model, impression-regularized finetuning achieved macro-F1 of 0.909 with high precision and recall, although the magnitude of improvement varied across model families and training settings. Per-level analysis further supported this aggregate result, showing high precision and recall across OCC and C1–C7, with relatively lower estimates clustered around the mid-cervical levels C3–C5. The largest benefits appeared in smaller models and low-data regimes, indicating that impression regularization can partially mitigate limitations in model scale and labeled data availability.

The Phi-3.5-Mini results further show that base-model choice matters for this task. Supplementary diagnostics indicated that Phi-3.5-Mini’s strong zero-shot performance was driven mainly by false-positive control rather than higher sensitivity (Table O in S1 Appendix). LoRA-based adaptation improved Phi-3.5-Mini relative to zero-shot, but standard LoRA and impression-regularized LoRA reached similar macro-F1 through different precision–recall profiles. A supplementary MediPhi-Instruct sensitivity analysis suggests that clinical-domain adaptation alone does not explain Phi-3.5-Mini’s zero-shot behavior: MediPhi-Instruct started lower but benefited from task-specific finetuning, including a modest additional gain from impression regularization. At the report level, however, F1 remained essentially unchanged (0.910 vs. 0.909), while the number of missed positive reports increased slightly (94.0 vs. 98.0). Because these are released-checkpoint comparisons rather than controlled ablations, we cannot causally separate pretraining data composition, architecture/tokenization, instruction tuning, model merging, clinical-domain adaptation, or adapter capacity. For practitioners, candidate base models should be evaluated both before and after the intended finetuning procedure, with attention to false-positive and false-negative profiles under the target label definition.

Compared with earlier NLP approaches, these findings suggest that high-quality labeling for this vertebral-level fracture extraction task does not require very large models or massive expert-annotated corpora. Prior supervised systems for spine pathology and vertebral compression fractures often required thousands to tens of thousands of labeled reports to reach strong performance [14,15]. For example, a multi-task BERT model trained on 1,578 cervical spine MRI reports reached macro-F1 0.62–0.83 [14], and a semantic text-mining system used 27,401 reports to achieve AUROC 0.98 [15], underscoring the substantial annotation burden of traditional methods. Closed, API-hosted LLMs can reduce this burden but raise privacy and governance concerns for protected health information [32]. More recently, open-weight LLMs have extracted spinal fracture information with less supervision but often rely on large models and complex prompting, such as Llama-3.1-70B with chain-of-thought prompting achieving F1 0.91 [13] and Zephyr-7B with tailored chain-of-thought and few-shot guidance reaching 94% accuracy on 890 CT reports [16], yet still face constraints of model size, intricate prompt design, limited label sets, and small, temporally narrow cohorts [12,17,18]. In this context, our results show that task-finetuned 3–4B open-weight models can approach the zero-shot performance of much larger LLMs while remaining practical for on-premises deployment in automated cohort curation, quality assurance, and monitoring pipelines. The measured hardware footprint suggests technical feasibility for workstation- or server-based on-premises use for retrospective cohort curation, quality assurance, and monitoring workflows.

Although the system is not intended to generate clinical impressions or replace radiologist judgment, accurate structured fracture labels from routine reports may support radiology workflows such as (i) radiologist-facing workflow support (e.g., worklist flags for predefined high-risk injury patterns to prompt expedited review and initiation of existing critical-result communication pathways), (ii) quality assurance and operational monitoring (e.g., reporting completeness, protocol adherence, and longitudinal trend dashboards), (iii) building trauma registries and research cohorts, and (iv) generating labels for training and monitoring downstream imaging models. For these use cases, the intended output is a compact report-derived structured label rather than a clinician-facing explanation or replacement impression. Accordingly, the deployed model returns deterministic JSON labels, which are easier to validate, store, integrate, and monitor than additional free-text outputs that may be verbose, variable, or difficult to control, reflecting an explicit trade-off between visible intermediate output and operational simplicity. This simplicity at the output level does not by itself ensure safe use: acting on the labels still carries practical risks such as false-alert burden, alert fatigue, and disruption to established workflows. The model is therefore best positioned as a non-blocking adjunct to existing review pathways rather than a primary safety mechanism, supported by validation against the source report, local performance auditing, prospective evaluation of alert thresholds, review of higher-risk cases, and schema updates as reporting practices evolve.

The targeted impressions used in this study are synthetic, self-generated summaries produced by the same instruction-tuned base model, not manually authored clinical impressions. They are prompted to mirror the language and diagnostic style of radiology impressions while emphasizing fracture-relevant conclusions, and they represent a constrained subset of impression content intended to isolate information most directly supporting structured label extraction. This design is particularly helpful for smaller models: direct report-to-JSON prediction requires simultaneously resolving nuanced clinical semantics (e.g., negation, temporality, and anatomical scope) and satisfying rigid structural constraints, which can be brittle in low-data regimes [33,34]. Synthetic targeted impressions provide a less format-constrained auxiliary task that reinforces fracture-relevant report semantics during training. We therefore treat them as latent auxiliary supervision rather than as clinical ground truth or inference-time explanations. This design can be viewed as a form of label-filtered self-distillation: the auxiliary objective trains the shared LoRA-adapted representation to reproduce the model’s own impressions, while the primary objective remains focused on structured JSON extraction. Selecting the candidate most consistent with the reference standard labels filters this supervision toward the extraction task, rather than treating all generated impressions as equally informative. Because these targets are generated once and held fixed, reproducing them anchors the representation to the model’s pretrained clinical-language behavior, discouraging drift under limited supervision while preserving single-pass deterministic inference. The sampling-and-selection scheme parallels prior work using intermediate, verifiable signals to stabilize learning [30,31,35], while remaining within a conventional supervised finetuning framework.

Error analysis of majority-vote predictions for the impression-regularized Gemma-3-4B model provides insight into the remaining failure modes. Among 30 false-negative cases, most reflected either missed detections of explicitly described fractures (43%) or linguistically ambiguous descriptions such as hedged or equivocal wording (30%). Among 23 false-positive cases, most arose from degenerative, pathologic, or post-surgical findings that warrant clinical attention but fall outside the acute traumatic fracture schema. These patterns suggest that residual errors are driven primarily by ambiguous language and label-definition boundaries rather than by unstable or uninterpretable model behavior, and that many apparent errors reflect scope mismatches rather than true semantic failures. In practice, mitigation could focus on enriching future training sets with targeted hard-positive and hard-negative examples, particularly reports involving ambiguous fracture language, multilevel injury descriptions, or common nonacute mimics. These higher-risk cases also provide natural targets for human review, periodic audit, and future dataset refinement, which may reduce recurring errors while preserving the simplicity of single-pass extraction for routine reports.

Although this study focuses on acute cervical spine fracture labeling, the proposed impression-regularized framework is not inherently fracture-specific. The label-verbalization control also improved macro-F1 over standard LoRA (0.901 vs. 0.885; Table N in S1 Appendix), suggesting that label-aligned auxiliary supervision contributes to the observed benefit beyond the impression format itself. The observed benefit may therefore reflect label-selected, task-relevant auxiliary supervision. In this study, the targeted impression provides a clinically natural intermediate that concentrates fracture-relevant diagnostic content and may help reinforce negation, temporality, and anatomic scope, properties shared by many report-level radiology tasks. The same strategy could extend to other radiologic phenotypes (e.g., degenerative, neoplastic, infectious) to enable rapid cohort identification from routine reports. Tasks requiring only report-level conclusions or coarse spatial resolution may be especially well suited, whereas applications needing fine-grained localization or quantitative measurements may benefit less.

This work has several limitations. First, the intermediate targeted impressions are self-generated model outputs rather than independently annotated clinical summaries. Although the sampling-and-selection procedure encourages consistency with curated fracture labels, it may still propagate biases from the base model, local reporting conventions, or the training dataset. Thus, label-consistency selection should be viewed as a task-alignment step rather than a guarantee of unbiased intermediate supervision, and future work should explicitly characterize these effects across institutions and reporting styles. Second, impression sampling is performed in a single offline step. More sophisticated on-policy or iterative schemes that update intermediate supervision as the model evolves could potentially yield further improvements by continuously refining the impression pool. Third, this single-center study reflects local English-language reporting conventions and fracture schemas, and its performance in external institutional settings remains unknown. Although supplementary year-level audits characterized observable changes in cohort composition, report text, vocabulary overlap, and available DICOM metadata, these analyses do not isolate the effects of reporting-template changes, or radiologist turnover. Because the regularizing impressions are synthetic and selected for consistency with local labels, the method does not require a globally uniform impression template; nonetheless, differences in institutional phrasing, negation, anatomic referencing, case mix, or documentation style may affect model calibration, and expansion to other languages or taxonomies would require multilingual models or schema re-specification. Although impression-regularized LoRA is not tied in principle to a language or label schema, multi-institutional, prospective, and multilingual validation remains essential before deployment. Finally, the benefit of impression regularization diminishes with model size; larger models may require alternative strategies to achieve further gains.

Future work should evaluate impression-regularized finetuning across institutions, anatomic regions, modalities, languages, and label schemas, including multi-label injury taxonomies. External validation should assess performance and calibration under report-style domain shift, including differences in report structure, template use, institutional and radiologist phrasing patterns, and non-English reporting environments, and determine whether prompt templates, impression-selection criteria, or label definitions require local adaptation. Systematic comparison of targeted impressions with other intermediate tasks (e.g., guided paraphrasing or structured rationales) will help clarify which forms of auxiliary supervision are most effective for various clinical NLP tasks. Prospective deployment studies, where automated labelers are embedded in real-time monitoring pipelines for imaging AI, will be essential to assess operational impact, clinician trust, and safety.

## 4. Method

In this study, we investigate three paradigms for structured label extraction from radiology reports, including direct extraction, impression-guided extraction, and our proposed impression-regularized finetuning framework, as illustrated in Fig 3.

**Fig 3:**
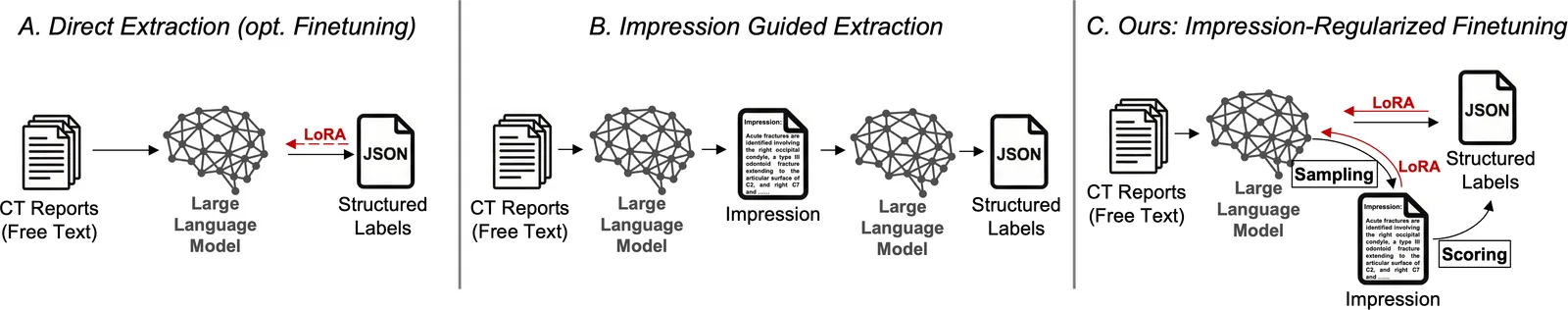
Overview of direct extraction, impression-guided extraction, and the proposed impression-regularized finetuning framework. **(A)** Direct extraction: a large language model (LLM) is optionally finetuned with LoRA to map CT reports directly to structured JSON labels. **(B)** Two-pass Impression-guided extraction: the LLM first generates a free-text radiology impression, which is then used as input to a second LLM call that produces JSON labels at inference time. **(C)** Impression-regularized finetuning (ours): during training, the LLM is adapted with LoRA to jointly predict JSON labels and an auxiliary impression, but only the JSON decoder is used at deployment.

### 4.1. Study cohort and data split

We constructed a retrospective cohort of all contrast and non-contrast enhanced cervical spine CT examinations performed between 2017 and 2022 in adult patients (≥18 years) presenting to the emergency department. Examinations were excluded if they were cancelled or incomplete, or if the corresponding radiology reports could not be retrieved from the Radiology Information System. No additional clinical, demographic, or report-content exclusions were applied. Each report was independently reviewed by a neuroradiologist (MP) and neurosurgery resident (CSL) at both the report-level and individual vertebral levels. Interrater agreement before adjudication was assessed using Cohen’s κ and was high at the report level (κ = 0.834) and individual anatomic levels (OCC, 0.731; C1, 0.836; C2, 0.880; C3, 0.809; C4, 0.762; C5, 0.748; C6, 0.827; and C7, 0.862). Discrepancies at either scope were subsequently adjudicated by a third reviewer (EC). To evaluate temporal generalization and data efficiency, reports from 2017 (n = 1,064) were used as the training/finetuning cohort, and reports from 2018–2022 (n = 7,503) were reserved as a fixed held-out temporal test cohort. In the primary full-data finetuning condition, all 1,064 reports from 2017 were used for supervised adaptation. The unit of analysis was the individual radiology report, corresponding to a single cervical spine CT encounter, consistent with the intended deployment setting in which each incoming report is labeled independently. Because acute fracture status is encounter-specific rather than a fixed patient-level attribute, patients with multiple eligible CT examinations could contribute more than one report. In total, 21 patients contributed multiple reports: 20 had negative labels across all encounters, and one patient imaged twice in 2019 had one fracture-positive and one fracture-negative study. Four patients had reports in both the 2017 training set and the 2018–2022 temporal test set; all four were fracture-negative, and their repeat examinations occurred years apart rather than representing duplicated reports.

Reports reflect routine clinical practice and vary in structure and scope. Cervical spine CT protocols routinely capture adjacent anatomy, including the skull base (occipital condyles to C1) and upper thoracic spine (to at least T1), leading to common mentions of head and thoracic structures. All report text was retained in full for modeling.

The final cohort comprised 8,567 reports with an overall report-level acute fracture prevalence of approximately 9.1%. Fracture prevalence was slightly higher in the training year (11.0%) compared to the test years (8.9%). The distribution of injuries was anatomically heterogeneous, with C7 (2.9%) and C2 (2.6%) being the most frequently affected levels. Detailed demographics and fracture statistics are summarized in Table 6. To characterize potential temporal distribution shift beyond fracture prevalence, we added supplementary year-level audits of cohort composition, report-text characteristics, vocabulary overlap, and available DICOM scanner/protocol metadata, comparing individual calendar years and the 2017 training year versus the pooled 2018–2022 temporal test period (Table A and Table B in S1 Appendix).

**Table 6:** Cohort Demographics and Fracture Distribution. Summary of report characteristics and fracture prevalence across the training (2017) and testing (2018–2022) sets. All counts are reported per radiology report (CT encounter); 21 patients imaged on more than one occasion each contribute more than one report. Abbreviations: OCC Occipital Condyle, SD Standard deviation.

Category	Statistic	Overall	Training (2017)	Testing (2018–2022)
Sample Size	Total Reports	8567	1064	7503
Age (years)	Mean ± SD	54.6 ± 21.8	51.2 ± 20.7	55.0 ± 21.9
	Range	18 - 101	18 - 96	18 - 101
Sex	Male	5504 (64.2%)	709 (66.6%)	4795 (63.9%)
	Female	3063 (35.8%)	355 (33.4%)	2708 (36.1%)
Fracture Status	Fracture	783 (9.1%)	117 (11.0%)	666 (8.9%)
	No Fracture	7784 (90.9%)	947 (89.0%)	6837 (91.1%)
Fracture Segment	OCC	144 (1.7%)	26 (2.4%)	118 (1.6%)
	C1	114 (1.3%)	19 (1.8%)	95 (1.3%)
	C2	222 (2.6%)	36 (3.4%)	186 (2.5%)
	C3	79 (0.9%)	8 (0.8%)	71 (0.9%)
	C4	94 (1.1%)	10 (0.9%)	84 (1.1%)
	C5	152 (1.8%)	18 (1.7%)	134 (1.8%)
	C6	184 (2.1%)	22 (2.1%)	162 (2.2%)
	C7	249 (2.9%)	32 (3.0%)	217 (2.9%)

### 4.2. Label definition and task scope

The task is vertebral-level classification of acute fractures at eight anatomical levels: occipital condyles (OCC) and C1–C7. Model outputs follow a fixed JSON schema with eight binary fields. A positive label indicates explicit support for an acute fracture at that level. A negative label indicates no acute fracture, including cases where:

- fractures are explicitly negated,
- the region is not mentioned, or
- fractures are described as chronic, healed, non-union, or otherwise non-acute.

Non-traumatic abnormalities and chronic fractures are therefore treated as negative for this task.

Training subsets of varying sizes (N=20,100,500,800,1064) were sampled from the 2017 data with stratification at the report level on overall fracture positivity, preserving the natural class distribution (prevalence = 11%). The 2018–2022 test set was fixed across all experiments.

### 4.3. Task formulation

Let x denote the Findings text of a radiology report, y the structured JSON label sequence encoding acute fracture status at OCC and C1–C7, and z a synthetic targeted impression summarizing only acute cervical spine fracture–relevant findings.

We use a single language model parameterized by θ, with one shared LoRA adapter [36]. Task-specific prompt templates define the following conditional distributions:

- JSON Extraction: $p_{\theta }\left(y|x\right)$
- Impression Generation: $p_{\theta }\left(z|x\right)$
- Impression-conditioned JSON extraction (used for scoring): $p_{\theta }\left(y|x,z\right)$

Given a report x, the goal is to predict the corresponding structured label sequence y. The targeted impression z is used only during training as an auxiliary latent variable.

### 4.4. Prompt design

We implement the task using a structured prompting framework consisting of:

- **System prompt** defining the model’s role as a radiologist specializing in cervical spine trauma.
- **JSON schema instruction** specifying key names, allowed values, and formatting constraints to ensure deterministic, machine-readable output.
- **Targeted impression prompt**, instructing the model to summarize only cervical spine fracture–relevant findings and explicitly exclude incidental or unrelated information.

Prompt templates were iteratively refined to minimize schema violations and stabilize behavior across model families and dataset sizes. Full prompt texts are provided in the S1 Appendix.

### 4.5. Baseline extraction approaches

We evaluate three baselines using the same prompt framework.

#### 4.5.1. Zero-shot extraction.

In the zero-shot condition, the instruction-tuned model directly generates the JSON labels from the report using the JSON schema prompt:


$$\begin{array}{c} {\hat{y}}_{\text{ZeroShot}}=\text{arg\textasciitilde{}}\underset{y}{\text{max}}p_{\theta }(y|x). \end{array}$$

No finetuning is performed; this baseline reflects out-of-the-box performance.

#### 4.5.2. Two-pass impression-guided Pipeline.

The two-pass baseline uses an explicit intermediate impression:

1. **Pass 1 (impression)**: generate a targeted impression from the report:

$$\tilde{z}\sim p_{\theta }(z|x).$$

2. **Pass 2 (JSON)**: Generate JSON labels conditioned on the report and its impression:

$$\begin{array}{c} {\hat{y}}_{\text{2}-\text{Pass}}=\text{arg\textasciitilde{}}\underset{y}{\text{max}}p_{\theta }(y|x,\tilde{z}). \end{array}$$

This design makes the intermediate impression step explicit but introduces a hard bottleneck. Any omission or mischaracterization in $\tilde{z}$ propagates directly to the labels and doubles inference-time computation.

#### 4.5.3. Standard LoRA finetuning.

LoRA adapts a pretrained model by freezing its original weights and learning a low-rank update ΔW=BA to selected weight matrices, where $B\in R^{d\times r}$, $A\in R^{r\times k}$ and rankr≪min(d,k) [36]. Only A and B are trained, reducing trainable parameters by several orders of magnitude, and because the base weights stay frozen the model retains its pretrained clinical-language knowledge while the learned update merges into the weights at deployment, leaving single-pass inference unchanged. Beyond this efficiency, restricting adaptation to a low-rank subspace constrains the capacity of the update and acts as an implicit regularizer, which is particularly advantageous in our low-data regimes (as few as 20–1,064 labeled reports). As a stronger baseline, we apply standard LoRA supervised finetuning to the direct mapping from report to JSON (x → y) [36]. The model is trained solely on the JSON extraction loss and behaves like the zero-shot extractor at inference, but with updated parameters.

### 4.6. Impression-regularized finetuning (Our Method)

We use synthetic self-generated targeted impressions to provide latent supervision during training without additional annotation (Fig 4). These impressions are prompted to mirror radiology impression style but constrained to acute cervical spine fracture findings. They are not treated as ground truth; instead, they are sampled from the model and selected by consistency with expert labels.

**Fig 4:**
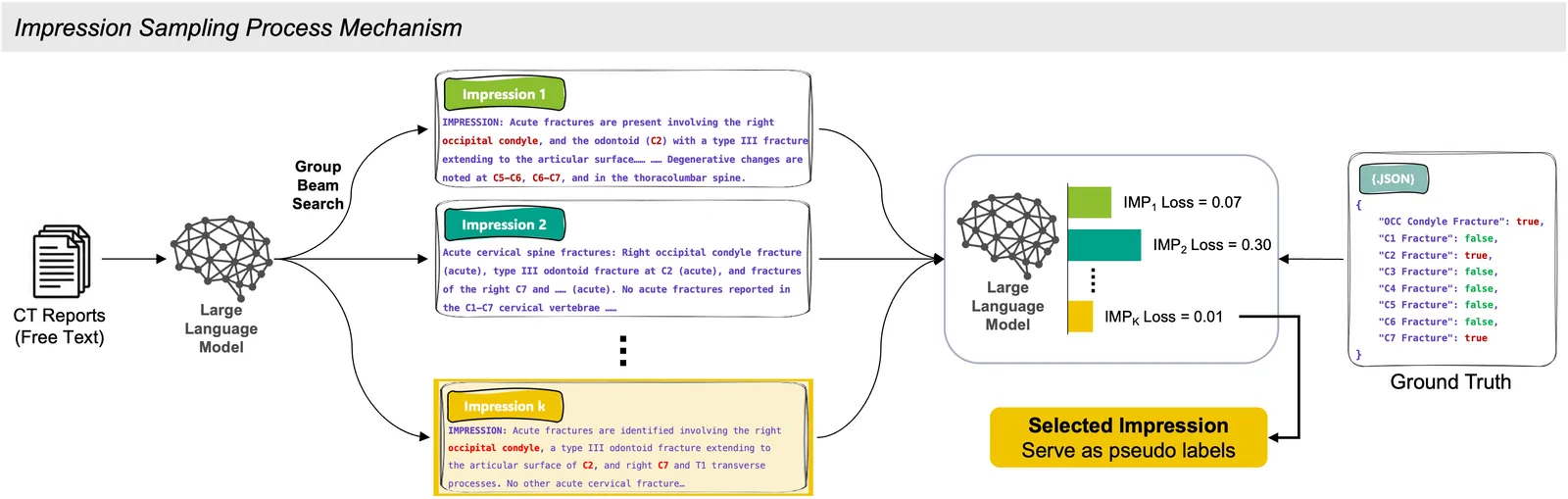
Impression sampling mechanism underlying impression-regularized finetuning. For each report, the LLM generates multiple candidate impressions via group beam search; each impression is scored by the loss of the resulting JSON prediction against ground-truth labels, and the lowest-loss impression is selected. These selected synthetic targeted impressions serve as pseudo-labels for the auxiliary impression loss used in the joint training objective.

#### 4.6.1. Impression sampling.

For each training example $\left({\text{x}}_{\text{i}},{\text{y}}_{\text{i}}\right)$, the model generates Kcandidate impressions using diverse group beam sampling [37] (here K=8 groups, two beams per group as suggested by Shi et al. [38]):


$$\begin{array}{c} Z_{\text{\textbackslash{}text\{\textbackslash{}specialsymfont 𝒾}}\}=\{z_{i\text{1}},\ldots ,z_{iK}\},\;\;z_{ij}\sim p_{\theta }\left(\cdot |x_{i}\right). \end{array}$$

Each candidate impression $z_{ij}$ is scored by the negative log-likelihood (NLL) of the reference standard JSON labels conditioned on both the report and the candidate impression:


$$\begin{array}{c} S_{ij}=-\text{log}p_{\theta }\left(y_{i}|x_{i},z_{ij}\right). \end{array}$$

We select the impression that makes the reference standard labels most likely:


$$\begin{array}{c} z_{i}^{*}=\text{arg}\underset{z_{ij}\in Z_{\text{\textbackslash{}text\{\textbackslash{}specialsymfont 𝒾}}}{\}}\text{min}S_{ij}. \end{array}$$

Even when candidates are lexically similar, small differences in negation, anatomic specificity, or acute-versus-chronic framing can materially affect label prediction; selecting $z_{i}^{*}$ therefore identifies a task-aligned intermediate summary that is most supportive of correct label extraction. As illustrated by the expert-constructed examples in Table C in S1 Appendix, the selected summaries align with standard cervical spine impressions, effectively distilling fracture-relevant information while suppressing unrelated content.

#### 4.6.2. Supervised finetuning with multi-task LoRA.

After selecting $z_{i}^{*}$, we finetune the model using multi-task LoRA supervised finetuning, jointly learning to generate the targeted impression and extract the JSON labels.

The primary JSON extraction loss is


$$\begin{array}{c} L_{json}=-\sum_{i}\text{log}p_{\theta }\left(y_{i}|x_{i}\right). \end{array}$$

The impression generation loss acts as an auxiliary regularizer:


$$\begin{array}{c} L_{imp}=-\sum_{i}\text{log}p_{\theta }\left(\overset{*}{\underset{i}{z}}|x_{i}\right)=\sum_{i}E_{z\sim q_{i}}\left[-\text{log}p_{\theta }\left(z|x_{i}\right)\right]. \end{array}$$

Up to an additive constant (the entropy of $q_{i}$, which is zero for a delta distribution), this auxiliary loss is equivalent to minimizing the KL divergence:


$$\begin{array}{c} L_{imp}=\sum_{i}\text{KL}\left(q_{i}(z,|\;p_{\theta }\left(z|x_{i}\right)\right). \end{array}$$

The total training objective is:


$$\begin{array}{c} L_{total}=L_{JSON}+\lambda L_{imp} \end{array}$$

An ablation on Gemma-3-4B varying λ∈{0,0.01,0.1,1.0} identifies λ=0.1 as achieving the best macro-F1 and report-level sensitivity, whereas smaller values behave like JSON-only finetuning and λ = 1.0 over-weights the impression loss and harms extraction (Table D in S1 Appendix). Intuitively, impression regularization encourages the model to reproduce intermediate summaries that best support correct labels. Because impressions are longer, less constrained outputs than JSON, this auxiliary task helps small models acquire domain vocabulary and report-level compositional structure while stabilizing optimization.

### 4.7. Model configuration and training details

We primarily evaluated small open-weight language models in the 3–4B parameter range, including Gemma-3-4B [39], Llama-3.2-3B [40], and Phi-3.5-Mini (4B) [41] accessed via Hugging Face. To assess scaling effects, we additionally evaluated Gemma-3 models with 1B, 12B, and 27B parameters [42]. Due to hardware limitations, the 27B-parameter Gemma-3 model was evaluated only in the zero-shot setting without finetuning. As a post hoc sensitivity analysis motivated by the Phi-3.5-Mini results, we evaluated MediPhi-Instruct [43], a clinical-domain derivative of Phi-3.5-mini-instruct; results are reported in Table O in S1 Appendix.

All finetuned models used LoRA, with adapters (rank = 8, α=16, dropout = 0.05) inserted into all linear layers [36] using the Hugging Face Transformers (v4.55.4) [44] and PEFT (v0.17.1) [45] libraries. Training used a learning rate of $\text{1}\times {\text{1}\text{0}}^{-\text{4}}$ and gradient norm clipping at 0.3. Effective batch sizes were 16 for standard JSON-only LoRA and 32 for impression-regularized LoRA to equalize the number of parameter update steps. In the primary setting, models were finetuned on the 2017 training cohort using a fixed 2-epoch LoRA schedule; a post hoc sensitivity analysis varying finetuning duration is reported in Fig B in S1 Appendix. All other hyperparameters followed default configurations and are listed in Table E in S1 Appendix.

All training and inference experiments were performed on a local workstation-class server within the institutional compute environment, using a single NVIDIA A100 80 GB PCIe GPU per run, with no model or data parallelism. LoRA finetuning was performed using float32 precision, whereas models were loaded in bfloat16 for inference. Experiments used local model weights and local report files; no report text was transmitted to external API services, and no cloud-hosted inference was used. For the primary Gemma-3-4B configuration, a representative two-epoch LoRA fine-tuning run on the 1,064-report 2017 development set required approximately 12 minutes and peaked at 49.5 GB VRAM. Smaller per-device batches can reduce peak memory while preserving the same effective batch size through additional gradient accumulation, with modest runtime overhead. At deployment, LoRA weights were merged into the base model, and inference used single-pass deterministic JSON decoding. In this on-premises single-node deployment setting, the Gemma-3-4B model processed reports in approximately 5 seconds at batch size 1 with less than 9 GB peak VRAM; batched inference further reduced amortized processing time to 0.71 seconds per report at batch size 24 with 27.7 GB peak VRAM. These latency and VRAM measurements therefore correspond to a single-node on-premises deployment model rather than a distributed, cloud-hosted, or edge-device architecture. In prospective deployment, the same model could be integrated as a server-side batch-processing or monitoring service connected to institutional report feeds, subject to local IT governance, access controls, and audit logging. Per-model inference and fine-tuning footprints are provided in Table F and Table G in S1 Appendix.

We also explored a two-pass LoRA variant in which the model was trained to generate an impression and then JSON labels conditioned on its own generated impression (schema in Fig C in S1 Appendix; preliminary results in Table H in S1 Appendix). In preliminary experiments this design did not provide consistent performance gains and increased inference cost, thus we focus on the single-pass multi-task configuration for all reported results.

### 4.8. Inference

At inference, all finetuned models produce JSON predictions using a single-pass direct extraction prompt without generating impressions:


$$\begin{array}{c} \hat{y}=\text{arg\textasciitilde{}}\underset{y}{\text{max}}p_{\theta }(y|x). \end{array}$$

Decoding is deterministic (greedy, temperature = 0) with a maximum of 512 new tokens to ensure reproducibility and support clinical audit and longitudinal monitoring.

### 4.9. Evaluation metrics and temporal stability analysis

All finetuning experiments were repeated with five independent random seeds. Metrics were computed per seed and reported as mean ± standard deviation. Primary evaluation uses macro-F1 across the eight vertebral categories (OCC and C1–C7). We also report macro-precision and macro-recall, and report-level performance where a case is considered positive if any level is positive; report-level metrics are summarized by sensitivity and specificity. Segment-level F1 scores for each vertebral level are provided in Table I and Table J in S1 Appendix, and complete per-level recall and precision for all model families and methods are provided in Table K in S1 Appendix.

To compare Impression-regularized LoRA with Standard LoRA on the macro-F1, we performed a hierarchical paired bootstrap (n=1000) over seeds and test cases. For each replicate b, we resampled *5* seeds with replacement; for each drawn seed pair, we then resampled test cases with replacement so that seeds and cases matched between methods, $\Delta_{b}={\bar{\text{F}\text{1}}}_{b}^{\;\text{Impression Regularized}}-{\bar{\text{F}\text{1}}}_{b}^{\;\text{Standard}}$. We report the mean observed difference $\Delta_{\text{obs}}$, 95% confidence intervals, and two-sided p-values estimated via bootstrap tail area: $p=\text{2}\times \text{min}(\text{Pr}(\Delta_{b}\geq \text{0}),\text{Pr}\left(\Delta_{b}\leq \text{0}\right))$ [46].

Temporal performance was assessed on the 2018–2022 test set using a 90-day sliding window shifted by 5 days, computing F1 per window. We characterized each model’s temporal trajectory along two complementary dimensions: its overall level, summarized by the mean F1 across windows, and its consistency, summarized by the standard deviation (SD) and coefficient of variation (CV) of F1 across windows. CV normalizes dispersion by the mean, allowing relative variability to be compared across models with different baseline performance; because CV is inflated when the mean is low, we report it alongside the absolute SD and mean rather than in isolation. These window-based summaries are descriptive: because the windows overlap substantially, the per-window values are autocorrelated, and the SD and CV should be interpreted as features of the performance trajectory rather than as estimates of independent sampling variability. The 90-day width was chosen to balance temporal resolution against the number of positive cases per window given the low per-level fracture prevalence; narrower windows contain too few positives for stable per-level F1, whereas wider windows over-smooth the trajectory. To confirm that the temporal results were not specific to this choice, we repeated the analysis with window widths of 30, 60, 90, 120, and 180 days (Table L in S1 Appendix). Because wider windows average over more reports, the SD and CV are smaller at larger window widths regardless of method, so only within-width comparisons are interpretable.

### 4.10. Error analysis

We focused error analysis on the Gemma-3-4B model finetuned with impression-regularized LoRA. At the report level, we characterized systematic failure modes using a majority-vote criterion, defining errors as cases where at least 3 of 5 models produced an incorrect report-level fracture prediction. This yielded 23 false-positive and 30 false-negative reports, which were reviewed by HML and AYC.

## Supporting information

S1 Appendix Detailed prompts, supplementary analyses, Table A-O, and Fig A-C. (DOCX)
