# Supplementary material for: Impression-regularized finetuning enables small language models to extract cervical spine fractures from radiology reports
Source: PLOS Digit Health. 2026 Oct 21;5(10):e0001786. doi: 10.1371/journal.pdig.0001786 (PMC13686323; doi:10.1371/journal.pdig.0001786)
Supplement: Supplementary file 1 [file pdig.0001786.s001.docx]

**Supplementary Materials**

Detailed Prompts used in this study

1. System prompt:

ROLE: You are an expert radiologist specializing in identifying *acute cervical spine fractures* from CT radiology reports.

TASK: Determine whether the report explicitly confirms or clearly negates an *acute* fracture involving any of the following regions:

1. Occipital condyles: OCC

2. Upper cervical vertebrae: C1 (atlas), C2 (axis, odontoid),

3. Lower cervical vertebrae: C3, C4, C5, C6, C7

INSTRUCTIONS:

1. Carefully analyze the report, focus on **acute** or **recent traumatic fracture**.

2. If a fracture is described as *old, remote, chronic, healing, healed,* or *nonunion*, treat it as no acute fracture.

3. If a region is **not mentioned** or the report **explicitly negates** a fracture, interpret it as no acute fracture.

4. Do not infer, interpret, or add information beyond what is explicitly stated in the report.

2. User prompt for JSON data generation:

````
<TASK:JSON> From the cervical spine CT radiology report, extract binary fracture labels for the regions.

OUTPUT RULES:
1. Provide output strictly as JSON with the specified keys only.
2. Ensure the JSON syntax is valid (no trailing commas, extra keys, or comments).
3. Assign binary labels as follows:
    - `true`: if an **acute** fracture is explicitly confirmed.
    - `false`: otherwise (negated, chronic, or not mentioned).

OUTPUT FORMAT:
Your JSON output must exactly match this template. Do not deviate from this format or infer information not explicitly mentioned.
```json
{
  "Occ_Condyle_Fracture": true/false,
  "C1_Fracture": true/false,
  "C2_Fracture": true/false,
  "C3_Fracture": true/false,
  "C4_Fracture": true/false,
  "C5_Fracture": true/false,
  "C6_Fracture": true/false,
  "C7_Fracture": true/false
}
```
````

3. User prompt for impression generation:

<TASK:IMP> From the cervical spine CT radiology report, generate a single, concise impression statement.

OUTPUT RULES:
1. Use clear, professional medical language suitable for a radiology impression.
2. Explicitly mention **acute or recent traumatic fractures** when present.
3. Clearly qualify **chronic**,** **healed,** **remote,** **old** or **nonunion** fractures if described.
4. State clearly if no acute fracture is described.
5. Report only explicit findings or clear negations. Do not infer or speculate.

Table A. **Cohort and report-text characteristics by calendar year.** Values are summarized per radiology report for the 2017 training year and each 2018–2022 temporal test year. Continuous variables are reported as mean ± SD and/or median (IQR), and categorical variables are reported as n (%). For age and report word count, p-values across all calendar years were calculated using Kruskal–Wallis tests, and p-values for 2017 versus pooled 2018–2022 were calculated using two-sided Mann–Whitney U tests. For female sex and report-level fracture status, p-values were calculated using chi-square tests, either across all calendar years or between 2017 and pooled 2018–2022. The number of reports and top-200 vocabulary Jaccard similarity were summarized descriptively and were not assigned p-values. Vocabulary overlap was calculated as the Jaccard similarity between the top 200 report terms in each calendar year and the top 200 report terms in 2017, after lowercasing, English stop-word removal, and filtering to alphabetic terms. Given the large sample size, p-values are intended to flag detectable temporal differences and should be interpreted together with the observed magnitudes.

| **Characteristic** | **2017** | **2018** | **2019** | **2020** | **2021** | **2022** | **p across years** | **p, 2017 vs 2018–2022** |
| --- | --- | --- | --- | --- | --- | --- | --- | --- |
| N reports | 1,064 | 1,344 | 1,524 | 1,259 | 1,539 | 1,837 | — | — |
| Age, years, mean ± SD | 51.2 ± 20.7 | 54.1 ± 21.8 | 53.5 ± 21.7 | 55.5 ± 21.9 | 55.6 ± 22.0 | 56.1 ± 22.1 | <0.001 | <0.001 |
| Female sex, n (%) | 355 (33.4) | 524 (39.0) | 553 (36.3) | 442 (35.1) | 544 (35.3) | 645 (35.1) | 0.087 | 0.089 |
| Any acute fracture, n (%) | 117 (11.0) | 114 (8.5) | 137 (9.0) | 120 (9.5) | 159 (10.3) | 143 (7.8) | 0.036 | 0.037 |
| Report word count, median (IQR) | 221 (157–303) | 212 (154–283) | 220 (164–294) | 236 (170–319) | 253 (188–349) | 233 (178–313) | <0.001 | <0.001 |
| Top-200 vocabulary Jaccard similarity vs 2017 | 1.000 | 0.896 | 0.869 | 0.717 | 0.646 | 0.667 | — | — |

Table B. **Selected DICOM scanner/protocol metadata audit for temporal distribution shift.** Categorical scanner/protocol metadata fields were compared across individual calendar years from 2017 to 2022 and between the 2017 training year and pooled 2018–2022 temporal test period using chi-square tests. Cramér’s V was used to quantify the magnitude of categorical distribution shift, with larger values indicating stronger association between metadata category and year or train-test group. Given the large sample size, p-values are intended to flag detectable temporal differences and should be interpreted together with Cramér’s V and the observed metadata distributions.

| **Metadata** | **Summary** | **Across-year shift, 2017–2022** | **Train-test shift, 2017 vs 2018–2022** | **Interpretation** |
| --- | --- | --- | --- | --- |
| Scanner manufacturer | GE Medical Systems in 100.0% of non-missing headers | V = 0.000 | V = 0.000 | No manufacturer change observed. |
| Scanner model | 3 models; Revolution CT most common, 73.7% | p < 0.001; V = 0.142 | p < 0.001; V = 0.103 | Small scanner-model mix shift. |
| Scanner software | 9 software-version labels | p < 0.001; V = 0.732 | p < 0.001; V = 0.686 | Large temporal software-version shift, consistent with scanner software updates. |
| Study/protocol label | 33 labels; top label 30.6% | p < 0.001; V = 0.140 | p < 0.001; V = 0.113 | Small protocol-label/study-description shift. |
| Body-part metadata | 6 labels; HEAD most common, 54.0% | p < 0.001; V = 0.089 | p < 0.001; V = 0.111 | Small shift; field may reflect combined head/cervical-spine trauma protocols or metadata conventions. |
| Reconstruction kernel | 3 labels; BONEPLUS most common, 54.1% | p < 0.001; V = 0.056 | p = 0.621; V = 0.011 | Minimal across-year shift and no meaningful train-test difference. |

Table C. Illustrative examples of candidate targeted-impression sampling and selection during impression-regularized training. The report examples shown here were constructed by a clinical expert (AYC) to demonstrate the impression-generation and selection procedure and do not correspond to individual patients or reports in the study dataset. Each constructed report was processed using the same sampling and scoring procedure used during training: Gemma-3-4B-it generated multiple candidate targeted impressions, and each candidate was scored by the negative log-likelihood of the reference structured fracture labels conditioned on the report and candidate impression (lower is better). The examples illustrate settings ranging from near-equivalent candidate impressions to substantial differences in label consistency, including cases involving acute multilevel fractures, acute and stable odontoid fractures, and no acute fracture. The candidate with the lowest score (highlighted) is selected as auxiliary supervision during impression-regularized training. Quantitative analysis of candidate-score variability in the actual 1,064-report training set is reported separately.

| Report | Sampled Impressions | Score |
| --- | --- | --- |
| HISTORY: 50-year-old. Trauma. Assess for cervical spine injury.  TECHNIQUE: CT cervical spine without intravenous contrast with multiplanar reformats.  COMPARISON: None available.  FINDINGS: Acute mildly displaced fractures of the C4, C5, and C6 spinous processes. No extension into the laminae. Possible minimal widening of the anterior C4-C5 and C5-C6 disc spaces. No facet joint widening or perched/dislocated facet. No significant vertebral translation or other acute traumatic malalignment. Vertebral body heights are maintained. No additional acute fracture identified. No large epidural hematoma within the limitations of CT. Mild prevertebral soft tissue swelling. Mild multilevel degenerative changes with small disc-osteophyte complexes, uncovertebral osteophytes, and facet arthropathy. No high-grade osseous spinal canal stenosis. | Impression: Acute mildly displaced fractures of the C4, C5, and C6 spinous processes are present. | 3.48E+00 |
|  | Impression: Acute mildly displaced fractures of the C4, C5, and C6 spinous processes are identified. | 3.50E+00 |
|  | Acute traumatic fractures are present at the C4, C5, and C6 spinous processes. No other acute fractures are identified. | 2.95E+00 |
|  | Acute traumatic fractures are present at the C4, C5, and C6 spinous processes. | 3.38E+00 |
|  | impression: Acute traumatic fractures are identified in segments of the lower cervical spine (C4-C6) involving the spinous processes, with possible minimal disc space widening. No other acute fractures are noted. | 1.24E+00 |
|  | impression: Acute traumatic fractures are identified in segments of the lower cervical spine (C4-C6) involving the spinous processes, with possible minimal disc space widening. No other acute fractures are described. | 1.24E+00 |
|  | Impression: Acute fractures of the spinous processes at C4, C5, and C6 are noted. | 1.22E+00 |
|  | Impression: Acute displaced fractures are noted at C4, C5, and C6 spinous processes. | 3.78E+00 |
| HISTORY: Trauma, rule out fracture  TECHNIQUE: CT images of the cervical spine were acquired without intravenous contrast.  COMPARISON: None available  FINDINGS: Acute type II odontoid fracture through the base of the dens with minimal distraction and fracture gap measuring up to 3 mm. The atlantoaxial lateral mass articulations remain congruent. Predental interval within normal limits. Atlantooccipital articulation is also congruent. Osseous canal at level fracture is capacious. Normal alignment of the remainder of the cervical spine. Multilevel moderate degenerative changes characterized by variable degrees of disc height loss, disc osteophyte complex, and facet arthropathy. No significant osseous canal stenosis. Multilevel foraminal stenosis noted most prominent at C5-6 where there is likely moderate to severe bilateral foraminal stenosis. Normal contour of the aerodigestive tract. No suspicious cervical adenopathy. Partially visualized lung apices are clear. | Impression: Acute type II odontoid fracture through the base of the dens with minimal distraction and fracture gap. | 1.43E-04 |
|  | Impression: Acute type II odontoid fracture with distraction is present. No other acute cervical spine fractures are identified. | 5.56E-05 |
|  | IMPRESSION: Acute type II odontoid fracture with distraction is present. No other acute cervical spine fractures are identified. | 7.08E-05 |
|  | CT CERVICAL SPINE IMPRESSION: Acute type II odontoid fracture with distraction is present. No other acute or recent traumatic fractures are identified. | 5.23E-05 |
|  | CT CERVICAL SPINE IMPRESSION: Acute type II odontoid fracture with distraction is present. No other acute cervical spine fractures are identified. | 4.73E-05 |
|  | IMPRESSION: Acute type II odontoid fracture through the base of the dens with minimal distraction and fracture gap is present. | 1.43E-04 |
|  | Impression: Acute type II odontoid fracture through the base of the dens with minimal distraction and fracture gap measuring up to 3 mm is present. | 1.28E-04 |
|  | Impression: Acute type II odontoid fracture through the base of the dens with minimal distraction and fracture gap measuring up to 3 mm is present. No other acute or recent traumatic fractures are described. | 1.61E-04 |
| HISTORY: Known type II odontoid fracture managed nonoperatively. Worsening upper extremity weakness. Assess for interval increase in odontoid fracture displacement.  TECHNIQUE: CT cervical spine without intravenous contrast with multiplanar reformats.  FINDINGS: Redemonstration of the known type II odontoid fracture. The degree and pattern of fracture fragment displacement and overlap are unchanged from the prior examination. Overall cervical spine alignment is unchanged. Severe central spinal canal stenosis is again noted and appears stable. | **IMPRESSION:** Stable findings with a known type II odontoid fracture; no acute or recent traumatic fractures are identified in the cervical spine. | 1.02E+00 |
|  | No acute fractures are described in this report. The report notes a stable type II odontoid fracture but focuses on its stability and lack of interval worsening. | 7.82E-01 |
|  | **TASK-IMPRESSION:** Stable findings with a known type II odontoid fracture; no acute or recent traumatic fractures are identified. | 1.08E+00 |
|  | Impression: Stable findings; no acute or recent traumatic fractures are described. | 1.56E-06 |
|  | No acute fracture is described. The report notes a stable type II odontoid fracture but focuses on its stability and lack of interval worsening. | 7.82E-01 |
|  | Impression: Stable findings with a known type II odontoid fracture; no acute or recent traumatic fractures are identified. | 1.05E+00 |
|  | No acute or recent traumatic fractures are described. The report notes a chronic type II odontoid fracture, but indicates stability in displacement. | 6.57E-01 |
|  | No acute fractures are described in the report. The report explicitly notes a stable type II odontoid fracture and stable cervical spine findings. | 8.60E-01 |
| HISTORY: Trauma, rule out fracture  TECHNIQUE: CT images of the cervical spine were acquired without intravenous contrast.  COMPARISON: None available  FINDINGS: No acute fracture or traumatic malalignment. Vertebral body heights are maintained. No destructive osseous lesion. No large epidural hematoma identified within the limitations of CT. Straightening of the normal cervical lordosis. Grade 1 anterolisthesis at C3-C4 and C4-C5, likely degenerative. Osseous fusion across the left C2-C3 facet joint. Multilevel degenerative changes, including endplate changes at C5-C6 and C6-C7. Sclerosis of the odontoid process is also favored degenerative. Advanced bilateral facet arthropathy at C3-C4 and C4-C5. No CT evidence of high-grade spinal canal stenosis. | No acute or recent traumatic fractures are identified. | 3.77E-06 |
|  | **IMPRESSION:** No acute or recent traumatic fractures are identified. | 1.30E-06 |
|  | IMPRESSION: No acute or recent traumatic fractures are identified. | 1.36E-06 |
|  | Impression: No acute or recent traumatic fractures are identified. | 1.21E-06 |
|  | IMPRESSION: No acute or recent traumatic fractures are identified. | 1.36E-06 |
|  | **IMPRESSION:** No acute or recent traumatic fractures are identified on this CT examination of the cervical spine. | 8.94E-07 |
|  | There is no acute fracture described in the report. | 8.97E-06 |
|  | There is no acute fracture described in this report. | 7.29E-06 |

Analysis of candidate-impression score variability across the 1,064-report training set revealed a highly skewed distribution, with a sharp increase around the 94th–95th percentile. More than 93% of reports exhibited very low candidate-score standard deviation (approximately 10⁻⁶–10⁻⁴), indicating that sampled impressions generally received nearly equivalent label-consistency scores and that candidate selection had little practical effect for most reports. Variability increased markedly in the upper tail, reaching 0.13 at the 95th percentile, indicating a small subset of reports for which alternative sampled impressions differed meaningfully in their consistency with the reference structured labels. Because random and score-based selection differ only in which sampled candidate is retained, the two approaches provide nearly identical supervision when candidate scores are similar. The benefit of score-based selection observed in Table N is therefore most plausibly concentrated among reports in this higher-variability subset. The expert-constructed examples in Table C are provided separately to illustrate how both low- and high-differentiation candidate sets can arise during the selection procedure.

Table D. Sensitivity analysis of the auxiliary impression loss weight (λ) for the Gemma-3-4B model, comparing standard LoRA (λ = 0) with impression-regularized training. Moderate weighting (λ = 0.1) yields the best overall performance, while over-weighting the auxiliary loss degrades extraction accuracy toward the baseline.

| **Method** | **Macro-F1** | **Macro Precision** | **Macro Recall** | **Report-level Sensitivity** | **Report-level Specificity** |
| --- | --- | --- | --- | --- | --- |
| Standard LoRA (λ=0.0) | 0.885 ± 0.022 | 0.883 ± 0.062 | 0.899 ± 0.028 | 0.937 ± 0.025 | 0.987 ± 0.010 |
| Ours (λ=0.01) | 0.904 ± 0.003 | 0.908 ± 0.039 | **0.903 ± 0.036** | 0.935 ± 0.032 | 0.994 ± 0.004 |
| Ours (λ=0.1) | **0.909 ± 0.004** | **0.929 ± 0.014** | 0.893 ± 0.010 | **0.943 ± 0.014** | **0.995 ± 0.002** |
| Ours (λ=1.0) | 0.889 ± 0.012 | 0.890 ± 0.054 | 0.892 ± 0.037 | 0.929 ± 0.041 | 0.993 ± 0.005 |

We varied the auxiliary impression loss weight λ ∈ {0, 0.01, 0.1, 1.0} for Gemma-3-4B. A small auxiliary weight improved macro-F1 and reduced variance relative to standard LoRA (λ = 0), with peak performance at λ = 0.1. Over-weighting the impression loss (λ = 1.0) reduced accuracy toward the baseline, so we use λ = 0.1 in all impression-regularized experiments (see §4.6.2).

Table E. **Finetuning schedules across training-set sizes.** All subsets were sampled without replacement from the 2017 cohort. For larger training sets (N ≥ 800), pilot experiments indicated convergence within two epochs, with additional epochs leading to overfitting; training was therefore limited to two epochs (100–134 optimization steps). For smaller training sets, the number of epochs was increased to maintain a broadly comparable number of optimization steps. Positive-case prevalence was approximately matched to that of the full cohort (11%–15%).

| **Train N** | **Epochs** | **Steps** | **Positive Cases in Training** | **Prevalence** |
| --- | --- | --- | --- | --- |
| 20 | 12 | 24 | 3 | 15% |
| 100 | 6 | 42 | 11 | 11% |
| 500 | 3 | 96 | 55 | 11% |
| 800 | 2 | 100 | 88 | 11% |
| 1064 | 2 | 134 | 117 | 11% |

Table F. Inference hardware footprint across evaluated models. Inference latency and VRAM footprint were measured on a single NVIDIA A100 80 GB PCIe GPU with **bfloat16** precision. Unless otherwise specified, results use batch size 1 and report model weight memory, peak inference VRAM, mean processing time per cervical-spine CT report, and generation throughput. The primary Gemma-3-4B-it model required 8.5 GB peak VRAM and processed reports in 4.65 seconds at batch size 1. In batched inference, the same model processed reports at an amortized 0.71 seconds per report with batch size 24, using 27.7 GB peak VRAM.

| **Model** | **Inference Batch Size** | **Weights VRAM** | **Peak VRAM** | **Mean s / report** | **Throughput tok/s** |
| --- | --- | --- | --- | --- | --- |
| Llama-3.2-3B | 1 | 6.0 GB | 6.6 GB | 1.94 | 44.8 |
| Phi-3.5-mini | 1 | 7.1 GB | 9.2 GB | 3.55 | 30.7 |
| Gemma-3-1B | 1 | 1.9 GB | 2.1 GB | 0.86 | 113.3 |
| Gemma-3-4B | 1 | 8.0 GB | 8.5 GB | 4.65 | 21.1 |
| Gemma-3-4B | 24 | 8.0 GB | 27.7 GB | 0.71 | 138.19 |
| Gemma-3-12B | 1 | 22.7 GB | 23.6 GB | 6.31 | 15.3 |
| Gemma-3-27B | 1 | 51.1 GB | 52.4 GB | 8.2 | 11.9 |

Table G. **LoRA fine-tuning hardware footprint across evaluated models.** LoRA fine-tuning memory was measured on a single NVIDIA A100 80 GB PCIe GPU using **float32** precision, maximum sequence length 1,280, and gradient checkpointing. The table reports the per-device micro-batch size and gradient-accumulation steps used to obtain the effective training batch size, trainable LoRA parameters relative to total model parameters, model weight memory, peak fine-tuning VRAM, and representative training time when measured. For Gemma-3-4B-it, smaller per-device micro-batches reduced peak VRAM from 49.5 GB to 24.3 GB while preserving the same effective batch size through additional gradient accumulation. Abbreviation: OOM = out of memory.

| **Model** | **Per device BS × Accum** | **Trainable Params / Total Params** | **Weights VRAM** | **Peak FT VRAM** | **Training Time (m)** |
| --- | --- | --- | --- | --- | --- |
| Llama-3.2-3B | 8 × 2 | 12.2 M / 3 225 M | 12.0 GB | 31.0 GB | 8.1 |
| Phi-3.5-mini | 8 × 2 | 14.9 M / 3 836 M | 14.3 GB | 22.1 GB | 10.9 |
| Gemma-3-1B | 16 × 1 | 6.5 M / 1006 M | 3.8 GB | 66.9 GB | 4.7 |
| Gemma-3-4B | 8 × 2 | 14.9 M / 3 895 M | 14.5 GB | 49.5 GB | 11.8 |
| Gemma-3-4B | 4 x 4 | 14.9 M / 3 895 M | 14.5 GB | 32.7 GB | 11.7 |
| Gemma-3-4B | 2 x 8 | 14.9 M / 3 895 M | 14.5 GB | 24.3 GB | 12.1 |
| Gemma-3-12B | 4 × 4 | 32.7 M / 11 799 M | 44.0 GB | 64.9 GB | 29.1 |
| Gemma-3-27B | 1 × 16 | — | (54.8 GB est.) | OOM | — |

Table H. Preliminary evaluation of a two-pass LoRA configuration for Llama-3.2-3B, in which the model first generates a targeted impression and then predicts JSON labels conditioned on that impression. Results (mean ± SD over multiple random seeds) are reported for macro-F1, macro-precision, macro-recall, and report-level sensitivity and specificity, alongside zero-shot, two-pass impression-guided zero-shot, standard LoRA, and single-pass impression-regularized LoRA baselines. The two-pass variant underperforms single-pass impression-regularized LoRA in macro-F1 while incurring substantially higher inference cost, so we did not pursue further tuning or full-scale evaluation.

| **Method** | **Macro-F1** | **Macro Precision** | **Macro Recall** | **Report-level Sensitivity** | **Report-level Specificity** |
| --- | --- | --- | --- | --- | --- |
| Zero-shot (Direct) | 0.791 | 0.747 | 0.856 | 0.889 | 0.983 |
| Zero-shot (Impression-guided) | 0.797 | 0.776 | 0.836 | 0.905 | 0.982 |
| LoRA (Standard) | 0.890 ± 0.004 | 0.918 ± 0.024 | 0.867 ± 0.021 | 0.918 ± 0.012 | 0.993 ± 0.003 |
| LoRA (Impression- Regularized) | **0.902 ± 0.004** | **0.937 ± 0.022** | **0.874 ± 0.020** | **0.924 ± 0.018** | **0.995 ± 0.003** |
| LoRA (2 Pass) | 0.863 ± 0.007 | 0.868 ± 0.010 | 0.862 ± 0.024 | 0.905 ± 0.017 | 0.989 ± 0.002 |

Table I. Performance of Gemma-3, Llama-3.2, and Phi-3.5 models for cervical fracture labeling under four regimes: zero-shot (direct), zero-shot (impression-guided), LoRA (Standard), and LoRA (impression-regularized). Reported metrics are macro-F1, macro-precision, macro-recall, report-level sensitivity and specificity, and F1 scores for occipital condyle (OCC) and C1–C7 levels; LoRA results are mean ± standard deviation across runs.

| **Model Variant** | **Method** | **Macro-F1** | **Macro Precision** | **Macro Recall** | **Report Level Sensitivity** | **Report Level Specificity** | **OCC F1** | **C1 F1** | **C2 F1** | **C3 F1** | **C4 F1** | **C5 F1** | **C6 F1** | **C7 F1** |
| --- | --- | --- | --- | --- | --- | --- | --- | --- | --- | --- | --- | --- | --- | --- |
| Gemma-3 | Zero-shot (Direct) | 0.669 | 0.543 | 0.932 | **0.968** | 0.934 | 0.454 | 0.700 | 0.783 | 0.567 | 0.554 | 0.685 | 0.745 | 0.862 |
|  | Zero-shot (Impression-guided) | 0.730 | 0.616 | **0.938** | **0.968** | 0.959 | 0.682 | 0.711 | 0.826 | 0.517 | 0.640 | 0.759 | 0.838 | 0.869 |
|  | LoRA (Standard) | 0.885 ± 0.022 | 0.883 ± 0.062 | 0.899 ± 0.028 | 0.937 ± 0.025 | 0.987 ± 0.010 | 0.756 ± 0.133 | **0.916 ± 0.007** | 0.936 ± 0.011 | **0.885 ± 0.025** | 0.892 ± 0.021 | 0.861 ± 0.016 | 0.912 ± 0.017 | 0.922 ± 0.009 |
|  | LoRA (Impression-regularized) | **0.909 ± 0.004** | **0.929 ± 0.014** | 0.893 ± 0.010 | 0.943 ± 0.014 | **0.995 ± 0.002** | **0.902 ± 0.038** | 0.915 ± 0.008 | **0.947 ± 0.007** | 0.880 ± 0.006 | **0.896 ± 0.004** | **0.882 ± 0.008** | **0.929 ± 0.011** | **0.924 ± 0.011** |
| Llama-3.2 | Zero-shot (Direct) | 0.791 | 0.747 | 0.856 | 0.889 | 0.983 | 0.794 | 0.854 | 0.837 | 0.656 | 0.726 | 0.790 | 0.822 | 0.846 |
|  | Zero-shot (Impression-guided) | 0.797 | 0.776 | 0.836 | 0.905 | 0.982 | 0.712 | 0.802 | 0.861 | 0.704 | 0.755 | 0.816 | 0.879 | 0.848 |
|  | LoRA (Standard) | 0.890 ± 0.004 | 0.918 ± 0.024 | 0.867 ± 0.021 | 0.918 ± 0.012 | 0.993 ± 0.003 | 0.843 ± 0.006 | 0.857 ± 0.027 | **0.928 ± 0.005** | 0.886 ± 0.019 | 0.886 ± 0.016 | 0.864 ± 0.011 | **0.929 ± 0.009** | 0.926 ± 0.006 |
|  | LoRA (Impression- regularized) | **0.902 ± 0.004** | **0.937 ± 0.022** | **0.874 ± 0.020** | **0.924 ± 0.018** | **0.995 ± 0.003** | **0.893 ± 0.014** | **0.865 ± 0.039** | 0.923 ± 0.009 | **0.899 ± 0.005** | **0.903 ± 0.014** | **0.885 ± 0.007** | 0.925 ± 0.014 | **0.929 ± 0.007** |
| Phi-3.5 | Zero-shot (Direct) | 0.859 | 0.885 | 0.845 | **0.898** | 0.992 | 0.790 | 0.852 | 0.826 | **0.857** | **0.876** | 0.853 | 0.914 | 0.902 |
|  | Zero-shot (Impression-guided) | 0.763 | **0.938** | 0.668 | 0.760 | 0.993 | 0.779 | 0.867 | 0.884 | 0.648 | 0.618 | 0.756 | 0.797 | 0.755 |
|  | LoRA (Standard) | 0.877 ± 0.008 | **0.938 ± 0.011** | 0.827 ± 0.018 | 0.880 ± 0.011 | **0.996 ± 0.000** | 0.843 ± 0.031 | **0.885 ± 0.008** | **0.923 ± 0.015** | 0.847 ± 0.004 | 0.847 ± 0.018 | 0.847 ± 0.015 | 0.912 ± 0.028 | 0.909 ± 0.010 |
|  | LoRA (Impression- regularized) | **0.880 ± 0.003** | 0.919 ± 0.004 | **0.848 ± 0.006** | 0.897 ± 0.004 | 0.995 ± 0.000 | **0.906 ± 0.004** | 0.871 ± 0.003 | 0.882 ± 0.004 | 0.826 ± 0.008 | 0.852 ± 0.004 | **0.857 ± 0.002** | **0.924 ± 0.006** | **0.923 ± 0.001** |

Table J. Gemma model scaling analysis for cervical fracture labeling across model sizes (1B, 4B, 12B, 27B) and methods: zero-shot (direct), LoRA (Standard), and LoRA (impression-regularized). Reported metrics are macro-F1, macro-precision, macro-recall, report-level sensitivity and specificity, and F1 scores for occipital condyle (OCC) and C1–C7 levels; LoRA results are mean ± standard deviation across runs.

| **Model Size** | **Method** | **Macro-F1** | **Macro Precision** | **Macro Recall** | **Report Level Sensitivity** | **Report Level Specificity** | **OCC F1** | **C1 F1** | **C2 F1** | **C3 F1** | **C4 F1** | **C5 F1** | **C6 F1** | **C7 F1** |
| --- | --- | --- | --- | --- | --- | --- | --- | --- | --- | --- | --- | --- | --- | --- |
| 1B | Zero-shot (Direct) | 0.215 | 0.480 | 0.158 | 0.183 | 0.990 | 0.245 | 0.000 | 0.166 | 0.311 | 0.190 | 0.341 | 0.291 | 0.177 |
|  | LoRA (Standard) | 0.646 ± 0.023 | 0.833 ± 0.017 | 0.553 ± 0.024 | 0.685 ± 0.030 | 0.990 ± 0.001 | 0.407 ± 0.163 | 0.601 ± 0.028 | 0.773 ± 0.038 | 0.653 ± 0.030 | 0.555 ± 0.045 | 0.668 ± 0.007 | 0.755 ± 0.009 | 0.751 ± 0.046 |
|  | LoRA (Impression- Regularized) | **0.782 ± 0.023** | **0.879 ± 0.028** | **0.713 ± 0.032** | **0.794 ± 0.025** | **0.994 ± 0.001** | **0.835 ± 0.028** | **0.766 ± 0.023** | **0.816 ± 0.008** | **0.700 ± 0.040** | **0.702 ± 0.072** | **0.744 ± 0.024** | **0.850 ± 0.034** | **0.840 ± 0.026** |
| 4B | Zero-shot (Direct) | 0.669 | 0.543 | **0.932** | **0.968** | 0.934 | 0.454 | 0.700 | 0.783 | 0.567 | 0.554 | 0.685 | 0.745 | 0.862 |
|  | LoRA (Standard) | 0.885 ± 0.022 | 0.883 ± 0.062 | 0.899 ± 0.028 | 0.937 ± 0.025 | 0.987 ± 0.010 | 0.756 ± 0.133 | **0.916 ± 0.007** | 0.936 ± 0.011 | **0.885 ± 0.025** | 0.892 ± 0.021 | 0.861 ± 0.016 | 0.912 ± 0.017 | 0.922 ± 0.009 |
|  | LoRA (Impression- Regularized) | **0.909 ± 0.004** | **0.929 ± 0.014** | 0.893 ± 0.010 | 0.943 ± 0.014 | **0.995 ± 0.002** | **0.902 ± 0.038** | 0.915 ± 0.008 | **0.947 ± 0.007** | 0.880 ± 0.006 | **0.896 ± 0.004** | **0.882 ± 0.008** | **0.929 ± 0.011** | **0.924 ± 0.011** |
| 12B | Zero-shot (Direct) | 0.901 | 0.903 | **0.907** | **0.925** | 0.994 | 0.873 | 0.846 | **0.942** | 0.903 | 0.886 | 0.902 | 0.939 | 0.919 |
|  | LoRA (Standard) | 0.921 ± 0.006 | 0.944 ± 0.016 | 0.900 ± 0.017 | 0.921 ± 0.025 | 0.996 ± 0.002 | 0.933 ± 0.008 | 0.908 ± 0.013 | **0.942 ± 0.009** | **0.912 ± 0.009** | 0.907 ± 0.011 | 0.902 ± 0.008 | 0.937 ± 0.004 | 0.924 ± 0.007 |
|  | LoRA (Impression- Regularized) | **0.925 ± 0.003** | **0.953 ± 0.008** | 0.899 ± 0.007 | 0.914 ± 0.008 | **0.998 ± 0.000** | **0.954 ± 0.012** | **0.910 ± 0.017** | 0.938 ± 0.006 | 0.908 ± 0.008 | **0.917 ± 0.007** | **0.903 ± 0.005** | **0.943 ± 0.006** | **0.926 ± 0.006** |
| 27B | Zero-shot (Direct) | 0.913 | 0.908 | 0.922 | 0.934 | 0.995 | 0.921 | 0.844 | 0.952 | 0.889 | 0.911 | 0.906 | 0.956 | 0.928 |

Table K. **Per-level precision and recall across all evaluated models and methods on the 2018–2022 test set.** For each anatomic level (OCC, C1–C7), precision (P) and recall (R) are reported under each method. Finetuned results (Standard LoRA and Impression-regularized LoRA) are means ± standard deviation across five seeds; zero-shot results are from a single deterministic run. "N/A" denotes an undefined precision at a level where the model made no positive predictions. Per-level positive counts are provided in Table 4. Abbreviations: OCC, occipital condyle; P, precision (PPV); R, recall (sensitivity).

| **Model** | **Method** | **OCC** | | **C1** | | **C2** | | **C3** | | **C4** | | **C5** | | **C6** | | **C7** | |
| --- | --- | --- | --- | --- | --- | --- | --- | --- | --- | --- | --- | --- | --- | --- | --- | --- | --- |
|  |  | **P** | **R** | **P** | **R** | **P** | **R** | **P** | **R** | **P** | **R** | **P** | **R** | **P** | **R** | **P** | **R** |
| Gemma-3-1B | Zero-Shot (Direct) | 0.607 | 0.144 | N/A | 0.000 | 0.895 | 0.091 | 0.328 | 0.296 | 0.344 | 0.131 | 0.411 | 0.291 | 0.552 | 0.198 | 0.688 | 0.101 |
|  | Standard LoRA | 0.869 ± 0.093 | 0.280 ± 0.144 | 0.853 ± 0.071 | 0.469 ± 0.057 | 0.873 ± 0.034 | 0.698 ± 0.070 | 0.771 ± 0.063 | 0.575 ± 0.071 | 0.877 ± 0.008 | 0.407 ± 0.047 | 0.694 ± 0.056 | 0.648 ± 0.034 | 0.827 ± 0.016 | 0.695 ± 0.009 | 0.896 ± 0.035 | 0.652 ± 0.077 |
|  | Impression-regularized LoRA | 0.830 ± 0.070 | 0.849 ± 0.065 | 0.801 ± 0.028 | 0.735 ± 0.040 | 0.907 ± 0.033 | 0.743 ± 0.015 | 0.853 ± 0.101 | 0.600 ± 0.059 | 0.899 ± 0.049 | 0.583 ± 0.098 | 0.861 ± 0.031 | 0.657 ± 0.039 | 0.941 ± 0.026 | 0.775 ± 0.038 | 0.939 ± 0.014 | 0.760 ± 0.043 |
| Gemma-3-4B | Zero-Shot (Direct) | 0.297 | 0.966 | 0.556 | 0.947 | 0.730 | 0.844 | 0.402 | 0.958 | 0.393 | 0.940 | 0.547 | 0.918 | 0.610 | 0.957 | 0.810 | 0.922 |
|  | Zero-Shot (Impression-Guided) | 0.518 | 1.000 | 0.565 | 0.958 | 0.799 | 0.855 | 0.356 | 0.944 | 0.482 | 0.952 | 0.654 | 0.903 | 0.745 | 0.957 | 0.812 | 0.935 |
|  | Standard LoRA | 0.687 ± 0.233 | 0.903 ± 0.065 | 0.956 ± 0.032 | 0.880 ± 0.030 | 0.938 ± 0.033 | 0.935 ± 0.024 | 0.874 ± 0.049 | 0.899 ± 0.012 | 0.897 ± 0.058 | 0.890 ± 0.026 | 0.886 ± 0.074 | 0.845 ± 0.059 | 0.904 ± 0.055 | 0.923 ± 0.027 | 0.926 ± 0.040 | 0.920 ± 0.029 |
|  | Impression-regularized LoRA | 0.905 ± 0.092 | 0.905 ± 0.035 | 0.954 ± 0.028 | 0.880 ± 0.034 | 0.950 ± 0.016 | 0.945 ± 0.015 | 0.862 ± 0.009 | 0.899 ± 0.012 | 0.922 ± 0.014 | 0.871 ± 0.010 | 0.939 ± 0.032 | 0.833 ± 0.014 | 0.945 ± 0.026 | 0.914 ± 0.014 | 0.955 ± 0.012 | 0.895 ± 0.027 |
| Gemma-3-12B | Zero-Shot (Direct) | 0.78 | 0.992 | 0.779 | 0.926 | 0.966 | 0.919 | 0.89 | 0.915 | 0.892 | 0.881 | 0.95 | 0.858 | 0.974 | 0.907 | 0.989 | 0.857 |
|  | Standard LoRA | 0.962 ± 0.032 | 0.907 ± 0.019 | 0.932 ± 0.025 | 0.886 ± 0.017 | 0.963 ± 0.014 | 0.923 ± 0.030 | 0.916 ± 0.023 | 0.910 ± 0.029 | 0.933 ± 0.021 | 0.883 ± 0.016 | 0.926 ± 0.031 | 0.881 ± 0.026 | 0.953 ± 0.013 | 0.922 ± 0.016 | 0.965 ± 0.018 | 0.887 ± 0.017 |
|  | Impression-regularized LoRA | 0.969 ± 0.017 | 0.941 ± 0.015 | 0.921 ± 0.030 | 0.899 ± 0.022 | 0.971 ± 0.009 | 0.906 ± 0.012 | 0.917 ± 0.019 | 0.899 ± 0.006 | 0.931 ± 0.015 | 0.902 ± 0.005 | 0.957 ± 0.007 | 0.855 ± 0.007 | 0.971 ± 0.003 | 0.917 ± 0.010 | 0.985 ± 0.004 | 0.873 ± 0.011 |
| Gemma-3-27B | Zero-Shot (Direct) | 0.866 | 0.983 | 0.767 | 0.937 | 0.952 | 0.952 | 0.877 | 0.901 | 0.906 | 0.917 | 0.951 | 0.866 | 0.974 | 0.938 | 0.975 | 0.885 |
| Llama-3.2-3B | Zero-Shot (Direct) | 0.760 | 0.831 | 0.844 | 0.853 | 0.896 | 0.785 | 0.525 | 0.873 | 0.602 | 0.917 | 0.768 | 0.813 | 0.768 | 0.877 | 0.810 | 0.885 |
|  | Zero-Shot (Impression-Guided) | 0.580 | 0.924 | 0.866 | 0.747 | 0.931 | 0.801 | 0.626 | 0.803 | 0.683 | 0.845 | 0.804 | 0.828 | 0.882 | 0.877 | 0.835 | 0.862 |
|  | Standard LoRA | 0.914 ± 0.030 | 0.783 ± 0.024 | 0.949 ± 0.013 | 0.783 ± 0.050 | 0.925 ± 0.020 | 0.931 ± 0.021 | 0.896 ± 0.040 | 0.879 ± 0.035 | 0.888 ± 0.043 | 0.886 ± 0.018 | 0.866 ± 0.038 | 0.864 ± 0.018 | 0.951 ± 0.033 | 0.910 ± 0.026 | 0.952 ± 0.013 | 0.900 ± 0.004 |
|  | Impression-regularized LoRA | 0.964 ± 0.026 | 0.832 ± 0.034 | 0.972 ± 0.012 | 0.781 ± 0.068 | 0.940 ± 0.019 | 0.906 ± 0.028 | 0.914 ± 0.031 | 0.885 ± 0.021 | 0.911 ± 0.022 | 0.895 ± 0.010 | 0.888 ± 0.038 | 0.884 ± 0.026 | 0.938 ± 0.024 | 0.912 ± 0.015 | 0.967 ± 0.036 | 0.895 ± 0.033 |
| Phi-3.5-Mini | Zero-Shot (Direct) | 0.690 | 0.924 | 0.781 | 0.937 | 0.904 | 0.763 | 0.919 | 0.803 | 0.971 | 0.798 | 0.915 | 0.799 | 0.947 | 0.883 | 0.959 | 0.853 |
|  | Zero-Shot (Impression-Guided) | 0.698 | 0.881 | 0.918 | 0.821 | 0.962 | 0.817 | 1.000 | 0.479 | 0.974 | 0.452 | 0.988 | 0.612 | 0.991 | 0.667 | 0.971 | 0.618 |
|  | Standard LoRA | 0.929 ± 0.026 | 0.773 ± 0.043 | 0.865 ± 0.016 | 0.905 ± 0.000 | 0.933 ± 0.006 | 0.914 ± 0.028 | 0.951 ± 0.018 | 0.763 ± 0.012 | 0.956 ± 0.017 | 0.762 ± 0.035 | 0.928 ± 0.017 | 0.781 ± 0.029 | 0.975 ± 0.009 | 0.858 ± 0.047 | 0.966 ± 0.008 | 0.858 ± 0.022 |
|  | Impression-regularized LoRA | 0.921 ± 0.001 | 0.892 ± 0.007 | 0.826 ± 0.008 | 0.920 ± 0.006 | 0.913 ± 0.002 | 0.854 ± 0.007 | 0.884 ± 0.004 | 0.775 ± 0.017 | 0.956 ± 0.010 | 0.769 ± 0.011 | 0.933 ± 0.004 | 0.793 ± 0.003 | 0.960 ± 0.009 | 0.891 ± 0.007 | 0.957 ± 0.003 | 0.891 ± 0.003 |

Table L. Window-size sensitivity of temporal performance (2018–2022). Per-window macro-F1 (mean ± SD across windows, averaged over five seeds) for sliding windows of 30–180 days (5-day step); zero-shot baselines shown for reference. Bold marks the highest mean per model and width. Impression-regularized LoRA gave the highest mean temporal F1 for every model at all widths; SD/CV shrink at wider windows by averaging, so only within-width comparisons are interpretable.

| **Model** | **Method** | **30-Day Temp F1** | **60-Day Temp F1** | **90-Day Temp F1** | **120-Day Temp F1** | **180-Day Temp F1** |
| --- | --- | --- | --- | --- | --- | --- |
| Gemma-3-1B | Zero-Shot (Direct) | 0.152 ± 0.102 | 0.185 ± 0.089 | 0.199 ± 0.074 | 0.206 ± 0.062 | 0.214 ± 0.046 |
|  | LoRA (Standard) | 0.496 ± 0.158 | 0.585 ± 0.124 | 0.615 ± 0.100 | 0.627 ± 0.084 | 0.638 ± 0.058 |
|  | LoRA (Impression-regularized) | **0.620 ± 0.161** | **0.720 ± 0.116** | **0.750 ± 0.084** | **0.763 ± 0.064** | **0.772 ± 0.044** |
| Llama-3.2-3B | Zero-Shot (Direct) | 0.669 ± 0.161 | 0.758 ± 0.108 | 0.780 ± 0.083 | 0.788 ± 0.063 | 0.797 ± 0.035 |
|  | Zero-Shot (Impression-guided) | 0.668 ± 0.166 | 0.752 ± 0.112 | 0.777 ± 0.082 | 0.787 ± 0.059 | 0.798 ± 0.031 |
|  | LoRA (Standard) | 0.732 ± 0.163 | 0.844 ± 0.094 | 0.872 ± 0.063 | 0.882 ± 0.043 | 0.888 ± 0.025 |
|  | LoRA (Impression-regularized) | **0.744 ± 0.162** | **0.858 ± 0.091** | **0.886 ± 0.062** | **0.896 ± 0.044** | **0.902 ± 0.028** |
| Phi-3.5-Mini | Zero-Shot (Direct) | 0.703 ± 0.161 | 0.807 ± 0.093 | 0.832 ± 0.069 | 0.843 ± 0.047 | 0.852 ± 0.025 |
|  | Zero-Shot (Impression-guided) | 0.611 ± 0.169 | 0.714 ± 0.112 | 0.739 ± 0.083 | 0.749 ± 0.065 | 0.758 ± 0.048 |
|  | LoRA (Standard) | 0.711 ± 0.166 | 0.820 ± 0.101 | 0.849 ± 0.074 | 0.861 ± 0.052 | 0.871 ± 0.027 |
|  | LoRA (Impression-regularized) | **0.720 ± 0.168** | **0.828 ± 0.102** | **0.854 ± 0.074** | **0.865 ± 0.052** | **0.873 ± 0.030** |
| Gemma-3-4B | Zero-Shot (Direct) | 0.599 ± 0.131 | 0.656 ± 0.082 | 0.671 ± 0.067 | 0.677 ± 0.055 | 0.681 ± 0.044 |
|  | Zero-Shot (Impression-guided) | 0.644 ± 0.127 | 0.708 ± 0.085 | 0.722 ± 0.069 | 0.728 ± 0.058 | 0.731 ± 0.044 |
|  | LoRA (Standard) | 0.738 ± 0.156 | 0.846 ± 0.089 | 0.871 ± 0.062 | 0.879 ± 0.045 | 0.885 ± 0.030 |
|  | LoRA (Impression-regularized) | **0.753 ± 0.162** | **0.867 ± 0.089** | **0.893 ± 0.055** | **0.901 ± 0.037** | **0.907 ± 0.024** |
| Gemma-3-12B | Zero-Shot (Direct) | 0.748 ± 0.165 | 0.859 ± 0.086 | 0.887 ± 0.059 | 0.895 ± 0.043 | 0.900 ± 0.028 |
|  | LoRA (Standard) | 0.760 ± 0.165 | 0.877 ± 0.086 | 0.904 ± 0.056 | 0.912 ± 0.037 | 0.918 ± 0.021 |
|  | LoRA (Impression-regularized) | **0.761 ± 0.168** | **0.881 ± 0.087** | **0.909 ± 0.057** | **0.917 ± 0.038** | **0.922 ± 0.021** |
| Gemma-3-27B | Zero-Shot (Direct) | **0.763 ± 0.165** | **0.874 ± 0.086** | **0.896 ± 0.053** | **0.904 ± 0.035** | **0.911 ± 0.022** |

Table M. Macro-F1 comparison between Impression- Regularized LoRA and Standard LoRA across training sizes. Values are mean ± SD over five random seeds. $\Delta_{\text{Obs}}$denotes the observed seed-averaged difference (Impression-regularized − Standard) reported as $\Delta_{\text{Obs}}\text{ }[95\% Confidence Intervals$] from a hierarchical paired bootstrap over seeds and test cases. Two-sided p-values are bootstrap tail areas; significance codes: * $p<0.05$, ** $p<0.01$, *** $p<0.001$, ns not significant.

| **Model** | **Training Data Size** | **Impression-** **Regularized LoRA** | **Standard LoRA** | **Δ (Obs)** | **P-value** | **Sig** |
| --- | --- | --- | --- | --- | --- | --- |
| gemma-3-1b | 20 | 0.309 ± 0.022 | 0.272 ± 0.038 | 0.037 [0.007, 0.080] | 0.004 | ** |
|  | 100 | 0.483 ± 0.121 | 0.337 ± 0.103 | 0.147 [0.101, 0.193] | <0.001 | *** |
|  | 500 | 0.647 ± 0.061 | 0.494 ± 0.081 | 0.153 [0.101, 0.193] | <0.001 | *** |
|  | 800 | 0.720 ± 0.029 | 0.587 ± 0.036 | 0.133 [0.083, 0.177] | <0.001 | *** |
|  | 1064 | 0.782 ± 0.023 | 0.646 ± 0.023 | 0.136 [0.104, 0.167] | <0.001 | *** |
| Llama_3_2-3B | 20 | 0.829 ± 0.006 | 0.810 ± 0.016 | 0.018 [0.001, 0.037] | 0.046 | * |
|  | 100 | 0.845 ± 0.009 | 0.826 ± 0.023 | 0.018 [0.004, 0.035] | 0.018 | * |
|  | 500 | 0.879 ± 0.010 | 0.857 ± 0.018 | 0.023 [0.006, 0.045] | 0.002 | ** |
|  | 800 | 0.891 ± 0.008 | 0.870 ± 0.014 | 0.021 [0.009, 0.033] | <0.001 | *** |
|  | 1064 | 0.902 ± 0.004 | 0.890 ± 0.004 | 0.012 [0.005, 0.020] | 0.002 | ** |
| gemma-3-4b | 20 | 0.798 ± 0.007 | 0.739 ± 0.023 | 0.059 [0.041, 0.075] | <0.001 | *** |
|  | 100 | 0.853 ± 0.035 | 0.797 ± 0.043 | 0.056 [0.014, 0.110] | <0.001 | *** |
|  | 500 | 0.889 ± 0.010 | 0.860 ± 0.020 | 0.029 [0.012, 0.048] | <0.001 | *** |
|  | 800 | 0.899 ± 0.001 | 0.876 ± 0.014 | 0.023 [0.011, 0.035] | <0.001 | *** |
|  | 1064 | 0.909 ± 0.004 | 0.885 ± 0.022 | 0.024 [0.007, 0.044] | 0.002 | ** |
| phi-3_5-mini | 20 | 0.861 ± 0.003 | 0.860 ± 0.009 | 0.001 [-0.007, 0.010] | 0.812 | ns |
|  | 100 | 0.867 ± 0.004 | 0.863 ± 0.002 | 0.004 [-0.001, 0.009] | 0.158 | ns |
|  | 500 | 0.872 ± 0.001 | 0.868 ± 0.005 | 0.004 [-0.002, 0.011] | 0.162 | ns |
|  | 800 | 0.876 ± 0.003 | 0.871 ± 0.005 | 0.006 [-0.001, 0.013] | 0.104 | ns |
|  | 1064 | 0.880 ± 0.003 | 0.877 ± 0.008 | 0.003 [-0.005, 0.012] | 0.432 | ns |
| gemma-3-12b | 20 | 0.900 ± 0.002 | 0.898 ± 0.002 | 0.002 [-0.001, 0.005] | 0.254 | ns |
|  | 100 | 0.907 ± 0.005 | 0.903 ± 0.009 | 0.004 [-0.004, 0.013] | 0.396 | ns |
|  | 500 | 0.917 ± 0.005 | 0.913 ± 0.008 | 0.004 [-0.003, 0.010] | 0.256 | ns |
|  | 800 | 0.923 ± 0.003 | 0.919 ± 0.007 | 0.004 [-0.004, 0.013] | 0.306 | ns |
|  | 1064 | 0.925 ± 0.003 | 0.921 ± 0.006 | 0.004 [-0.003, 0.012] | 0.254 | ns |

Table N. Ablation study on impression selection strategies using the Gemma-3-4B model with an impression weight of λ = 0.1. Panel A. Performance comparison between Standard LoRA finetuning, random impression sampling, label verbalization, and score-based selection.

| **Method** | **Macro-F1** | **Macro Precision** | **Macro Recall** | **Report-level Sensitivity** | **Report-level Specificity** |
| --- | --- | --- | --- | --- | --- |
| Standard LoRA (Baseline) | 0.885 ± 0.022 | 0.883 ± 0.062 | 0.899 ± 0.028 | 0.937 ± 0.025 | 0.987 ± 0.010 |
| Random Sampling | 0.895 ± 0.017 | 0.885 ± 0.035 | 0.910 ± 0.009 | 0.954 ± 0.009 | 0.990 ± 0.005 |
| Label Verbalization | 0.901 ± 0.005 | 0.951 ± 0.015 | 0.859 ± 0.020 | 0.885 ± 0.018 | 0.998 ± 0.001 |
| Ours (score-based selection) | 0.909 ± 0.004 | 0.929 ± 0.014 | 0.893 ± 0.010 | 0.943 ± 0.014 | 0.995 ± 0.002 |

Panel B. Paired comparisons against the proposed score-based selection method are reported as the observed seed-averaged difference in macro-F1 (Δ_Obs_, Ours - Method) with 95% confidence intervals from a hierarchical paired bootstrap over seeds and test cases. Two-sided p-values are bootstrap tail areas; significance codes: * p < 0.05, ** p < 0.01.

| **Comparison** | **Δ Macro-F1 (95% CI), Ours - Method** | **P-value** | **sig** |
| --- | --- | --- | --- |
| Standard LoRA vs. Ours | 0.024 [0.007, 0.044] | 0.002 | ** |
| Random Sampling vs. Ours | 0.014 [0.000, 0.031] | 0.042 | * |
| Label Verbalization vs. Ours | 0.008 [0.001, 0.015] | 0.040 | * |

To separate the effects of impression generation, direct label-aligned auxiliary supervision, and impression selection, we compared standard LoRA finetuning with three auxiliary variants: random sampling, label verbalization, and score-based selection. The random baseline selected impressions uniformly from the same candidate pool used by the proposed method. The label-verbalization control replaced the synthetic targeted impression with a plain-text statement generated directly from the reference standard JSON labels, listing positive and negative vertebral levels without clinical summarization or reasoning. This non-impression control was included to test whether the gains could be explained by adding an auxiliary supervised output aligned with the labels, rather than by using an impression-like intermediate representation.

Random sampling yielded modest gains over Standard LoRA (macro-F1 0.895 vs. 0.885), driven mainly by higher macro-recall (0.910 vs. 0.899) and improved report-level sensitivity (0.954 vs. 0.937). This suggests that generating domain-relevant intermediate text provides a weak regularization effect that helps recover borderline positive cases. However, precision remained essentially unchanged (0.885 vs. 0.883). Label verbalization also improved macro-F1 relative to Standard LoRA (0.901 vs. 0.885) and produced the highest macro-precision and report-level specificity among the auxiliary controls (0.951 and 0.998, respectively), but at the cost of lower macro-recall (0.859) and report-level sensitivity (0.885). This indicates a more conservative prediction profile and suggests that direct label-aligned auxiliary supervision contributes to performance but does not reproduce the recall behavior of impression-based auxiliary text.

In contrast, score-based selection achieved the strongest and most stable performance (macro-F1 0.909). By selectively reinforcing low-loss impressions, it improved macro-precision relative to random sampling (0.929 vs. 0.885) while preserving higher macro-recall and report-level sensitivity than label verbalization (0.893 vs. 0.859 and 0.943 vs. 0.885, respectively). These results indicate that filtering impressions by label consistency improves both accuracy and training stability, and that selected targeted impressions provide a more balanced precision–recall profile than direct label restatement alone.

This interpretation is further supported by likelihood analysis on the binary label tokens. Random sampling produced a mean cross-entropy loss of 0.080 (92.3% average probability on the correct token), whereas score-based selection reduced the loss to 0.048 (95.3%), corresponding to a 3.3% relative increase in confidence. Although the absolute change in token-level probability is small, such shifts might be sufficient to alter outcomes for borderline cases near the decision threshold. Together, these results indicate that the benefit of the proposed approach should not be attributed solely to impression generation or to the domain-native impression format alone, but rather to selected, task-relevant auxiliary supervision, with targeted impressions serving as an effective clinically natural representation in this setting.

Table O. **Phi-family error-profile diagnostics on the common 2018–2022 test set.** Phi-3.5-Mini and MediPhi-Instruct were evaluated under zero-shot prompting, standard LoRA, and impression-regularized LoRA on the shared 7,503-report test set. Macro metrics are computed across OCC and C1–C7; Total FN and Total FP denote summed label-level errors, and report-level metrics treat any report with at least one acute fracture label as positive. LoRA results are mean ± SD across five runs. Impression-regularized LoRA significantly improved MediPhi-Instruct macro-F1 over standard LoRA (p = 0.04). Abbreviations: SEN, sensitivity/recall; PPV, positive predictive value; FN, false negative; FP, false positive.

| Model | Method | Macro F1 | Macro Precision | Macro Recall | Total FN | Total FP | Report-Level F1 | Report-Level FN | Report-Level FP |
| --- | --- | --- | --- | --- | --- | --- | --- | --- | --- |
| Phi-3.5-Mini | Zero-shot | 0.859 | 0.885 | 0.845 | 168 | 122 | 0.907 | 68 | 55 |
|  | Standard LoRA | 0.877 ± 0.008 | 0.938 ± 0.011 | 0.827 ± 0.018 | 171.8 ± 19.3 | 56.8 ± 9.0 | 0.918 ± 0.006 | 80.0 ± 7.6 | 25.0 ± 1.6 |
|  | Impression-regularized LoRA | 0.880 ± 0.003 | 0.919 ± 0.004 | 0.848 ± 0.006 | 152.0 ± 5.5 | 75.0 ± 4.3 | 0.920 ± 0.002 | 68.4 ± 2.5 | 35.4 ± 1.1 |
| MediPhi-Instruct | Zero-shot | 0.730 | 0.905 | 0.638 | 385 | 88 | 0.817 | 183 | 33 |
|  | Standard LoRA | 0.869 ± 0.005 | 0.936 ± 0.013 | 0.813 ± 0.015 | 186.8 ± 14.1 | 55.2 ± 11.6 | 0.910 ± 0.004 | 94.0 ± 6.8 | 19.0 ± 3.7 |
|  | Impression-regularized LoRA | 0.881 ± 0.009 | 0.956 ± 0.008 | 0.823 ± 0.021 | 182.6 ± 22.9 | 39.8 ± 8.1 | 0.909 ± 0.008 | 98.0 ± 12.5 | 15.0 ± 4.1 |

**Supplementary Section. Phi-3.5-Mini zero-shot strength and finetuning behavior.**

We performed additional diagnostics on the common 2018–2022 test set to clarify why Phi-3.5-Mini produced the strongest zero-shot baseline and why impression-regularized LoRA added little over standard LoRA. Phi-3.5-Mini’s zero-shot advantage was not explained by higher sensitivity. Gemma-3-4B had higher macro-recall than Phi-3.5-Mini (0.932 vs. 0.845) but much lower macro-precision (0.543 vs. 0.885), indicating substantially more false-positive overcalling (Tables I). Schema completion also did not explain the ranking, as Gemma-3-4B produced complete extracted outputs but had the lowest zero-shot macro-F1. Thus, Phi-3.5-Mini’s zero-shot strength appeared to reflect stronger false-positive control under the study label definition.

To examine whether this advantage reflected clinical-domain specialization, we evaluated MediPhi-Instruct, a Phi-3.5-mini-instruct derivative adapted to the medical/clinical domain, under the same prompts, LoRA configuration, impression-selection procedure, and test set. MediPhi-Instruct started lower than Phi-3.5-Mini in zero-shot macro-F1 (0.730 vs. 0.859) and was more conservative, with lower macro-recall (0.638). This suggests that clinical-domain adaptation alone does not account for Phi-3.5-Mini’s zero-shot behavior. However, MediPhi-Instruct is not a controlled ablation, because it also differs in training, model merging, and alignment procedures.

After finetuning, the two Phi-3.5-lineage checkpoints reached similar macro-F1 values of approximately 0.88 despite different zero-shot starting points. For Phi-3.5-Mini, standard LoRA and impression-regularized LoRA reached similar aggregate performance through different precision–recall profiles: standard LoRA further reduced false positives, whereas impression-regularized LoRA recovered more positive labels but introduced more false positives relative to standard LoRA. For MediPhi-Instruct, task-specific finetuning substantially improved recall from its conservative zero-shot profile, and impression-regularized LoRA provided a modest additional improvement over standard LoRA (0.869 to 0.881, p=0.04). These findings suggest that the benefit of impression regularization depends on the checkpoint’s initial error profile and should be assessed after the intended finetuning procedure rather than inferred from aggregate zero-shot F1 alone. Because these are released-checkpoint comparisons, they should be interpreted as behavioral diagnostics rather than causal evidence about pretraining data composition, architecture/tokenization, instruction tuning, model merging, clinical-domain adaptation, or adapter capacity.

Fig A. Temporal performance of fracture labelers across model families and adaptation strategies. For each model (rows; Gemma-3-1B/4B/12B, Llama-3.2 3B, Phi-3.5-Mini), we plot macro-F1 (left column) and report-fracture sensitivity (right column) over time on the 2018–2022 test set using 90-day sliding windows. Curves compare zero-shot (direct), zero-shot (impression-guided), LoRA (standard), and LoRA (impression-regularized) variants; shaded bands denote variability across independent runs. Method-colored dashed horizontal lines indicate each variant’s mean performance over the evaluation period. Dashed green lines show the contemporaneous prevalence of acute fractures. Impression-regularized LoRA generally yields higher and more temporally stable performance, particularly for smaller models.

Fig B. **Epoch-sensitivity analysis for Gemma-3-4B**. Macro-F1 on the fixed 2018–2022 temporal test cohort is shown across finetuning durations. Dark diamonds and the connecting line indicate the mean across available runs, and gray points show individual seed-level results with horizontal jitter for visualization. The zero-shot condition was evaluated once without finetuning. Epochs 1, 3, 4, 6, and 10 were evaluated with three seeds, while epoch 2 corresponds to the primary 2-epoch configuration evaluated with five seeds. Performance increased substantially after one epoch and was broadly stable from 2 to 10 epochs, with no clear upward trend after 2 epochs.

Fig C. Training schema for structured fracture extraction. Schema A depicts standard LoRA finetuning with report-to-JSON supervision only. Schema B illustrates the multi-pass design, in which impression generation and JSON extraction are trained within a single sequence using two prompts, resulting in a hard impression bottleneck at inference time. Schema C shows impression-regularized training, where impression generation is supervised as an auxiliary task during training, but JSON extraction is performed in a single pass at inference. Abbreviation: IMP: Impression
